# Advancing the Economic and Environmental Sustainability of the NEWgenerator Nonsewered Sanitation System

**DOI:** 10.1021/acsenvironau.3c00001

**Published:** 2023-05-05

**Authors:** Shion Watabe, Hannah A. C. Lohman, Yalin Li, Victoria L. Morgan, Lewis S. Rowles, Tyler Stephen, Hsiang-Yang Shyu, Robert A. Bair, Cynthia J. Castro, Roland D. Cusick, Daniel H. Yeh, Jeremy S. Guest

**Affiliations:** † Department of Civil and Environmental Engineering, University of Illinois Urbana-Champaign, 205 N. Mathews Avenue, Urbana, Illinois 61801, United States; ‡ Institute for Sustainability, Energy, and Environment, University of Illinois Urbana-Champaign, 1101 W. Peabody Dr., Urbana, Illinois 61801, United States; § Department of Civil and Environmental Engineering, University of South Florida, 4202 E. Fowler Avenue, Tampa, Florida 33620, United States

**Keywords:** nonsewered sanitation (NSS) system, QSDsan, multi-unit reinvented toilet (MURT), techno-economic analysis (TEA), life cycle assessment (LCA), decentralized sanitation, on-site sanitation

## Abstract

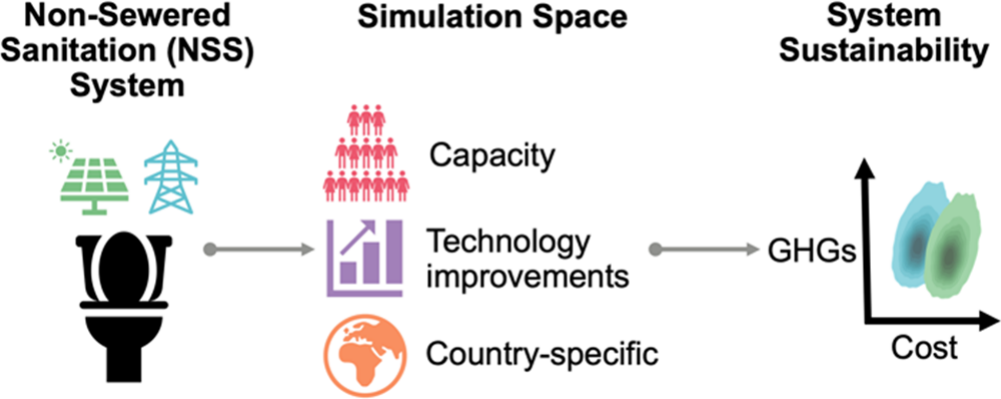

Achieving safely managed sanitation and resource recovery in areas that are rural, geographically challenged, or experiencing rapidly increasing population density may not be feasible with centralized facilities due to space requirements, site-specific concerns, and high costs of sewer installation. Nonsewered sanitation (NSS) systems have the potential to provide safely managed sanitation and achieve strict wastewater treatment standards. One such NSS treatment technology is the NEWgenerator, which includes an anaerobic membrane bioreactor (AnMBR), nutrient recovery via ion exchange, and electrochlorination. The system has been shown to achieve robust treatment of real waste for over 100 users, but the technology’s relative life cycle sustainability remains unclear. This study characterizes the financial viability and life cycle environmental impacts of the NEWgenerator and prioritizes opportunities to advance system sustainability through targeted improvements and deployment. The costs and greenhouse gas (GHG) emissions of the NEWgenerator (general case) leveraging grid electricity were 0.139 [0.113–0.168] USD cap^–1^ day^–1^ and 79.7 [55.0–112.3] kg CO_2_-equiv cap^–1^ year^–1^, respectively. A transition to photovoltaic-generated electricity would increase costs to 0.145 [0.118–0.181] USD cap^–1^ day^–1^ but decrease GHG emissions to 56.1 [33.8–86.2] kg CO_2_-equiv cap^–1^ year^–1^. The deployment location analysis demonstrated reduced median costs for deployment in China (−38%), India (−53%), Senegal (−31%), South Africa (−31%), and Uganda (−35%), but at comparable or increased GHG emissions (−2 to +16%). Targeted improvements revealed the relative change in median cost and GHG emissions to be −21 and −3% if loading is doubled (i.e., doubled users per unit), −30 and −12% with additional sludge drying, and +9 and −25% with the addition of a membrane contactor, respectively, with limited benefits (0–5% reductions) from an alternative photovoltaic battery, low-cost housing, or improved frontend operation. This research demonstrates that the NEWgenerator is a low-cost, low-emission NSS treatment technology with the potential for resource recovery to increase access to safe sanitation.

## Introduction

The United Nations has recognized safe sanitation as a universal right^1,2^ and set the goal to attain universal coverage of safe sanitation services by 2030.^3^ However, the world is not on track to achieve this target.^4^ One driving force influencing sanitation coverage is rapid global urbanization^5^ coupled with increasing population density in peri-urban areas, urban fringe areas, and informal settlements that lack conventional or safe sanitation infrastructure. Significant challenges exist for extending infrastructure services such as sewer, water, and grid electricity to these areas, which leaves low-resource and vulnerable populations at risk for unsafe sanitation. In 2020, it was reported that the use of onsite sanitation (e.g., septic tanks, improved latrines, decentralized wastewater systems) was just as common as sewered sanitation in these settings (43% population coverage by each).^4^ Despite its importance to communities, onsite sanitation is often constructed and operated with limited regulatory oversight and can experience high loadings (i.e., usage) which can overwhelm system capacity, posing a risk to local water resources and public health.^6^ Nonsewered sanitation (NSS) systems are prefabricated, integrated treatment units with the potential to meet strict wastewater treatment standards. NSS systems can be used to extend safe sanitation to rural areas where sewered connections are not feasible, in temporary or permanent settlements, and in locations with geographical challenges (e.g., flood-prone, high-water table, isolated areas).

The development of NSS systems has been accelerated in recent years, in part due to the Bill & Melinda Gates Foundation’s Reinvent the Toilet initiative targeting reliable performance, affordable costs, and low greenhouse gas (GHG) emissions.^7^ A range of technologies, serving individual households (single-unit reinvented toilets), clusters of households (multi-unit reinvented toilets), and even communities of tens of thousands of people (Omni Processors), have emerged to tackle the sanitation challenge.^8−18^ In recognition of the potential of NSS to contribute to safe sanitation coverage, the International Organization for Standardization (ISO) established testing and performance requirements (ISO 30500) for NSS systems. The stringent testing and performance requirements ensure human health protection in solid output and liquid effluent via log reduction values (LRV) of human enteric bacterial pathogens, thresholds for effluent chemical oxygen demand (COD), total suspended solids (TSS), and pH, as well as nutrient removal requirements for total nitrogen (TN) and total phosphorus (TP).^19^

One such NSS technology that has achieved robust treatment of real human waste streams (97.6 ± 3.1% TSS removal, 94.5 ± 5.0% COD removal, 7.4 ± 1.5% LRV, 82.1 ± 24.0% total nitrogen removal)^8^ is the NEWgenerator. The NEWgenerator is a NSS backend technology that can be integrated with various frontends (i.e., user interfaces such as urinal, squatting or seating pan, and associated plumbing) to serve 100+ users. The technology consists of an anaerobic membrane bioreactor (AnMBR), a nutrient capture system (NCS) with ion exchange and carbon sorption, and an electrochlorination (EC) unit.^8^ All NEWgenerator units are fully integrated and housed in a mini-shipping container (4.56 m^2^ footprint) for a compact, portable design. To date, the financial viability and GHG implications of this technology, as well as its sensitivities to site-specific considerations (i.e., contextual parameters such as the electricity mix),^20^ are yet to be quantified. As we seek to advance the sustainable provision of safe sanitation, a deeper understanding of the location-specific economic cost and life cycle environmental impacts is vital to prioritize investment in the research, development, and deployment of NSS systems.

The objectives of this study were (i) to elucidate the drivers governing the environmental and economic viability of the NEWgenerator and (ii) to prioritize opportunities to advance system sustainability through targeted improvements and prioritized deployment of the technology. To this end, we used a quantitative sustainable design (QSD)^20^ approach to characterize the technological, economic, and environmental sustainability of the NEWgenerator technology. Our analysis leveraged detailed design and published performance data from a 534-day field trial in an informal settlement in South Africa, treating high-strength blackwater and yellow water from a community toilet facility.^8^ In addition to the NEWgenerator, the analysis included a frontend, pretreatment, a foundation, and onsite sludge treatment to simulate the fully operational NSS system. We evaluated the system using QSDsan,^20^ an open-source, community-led platform for the QSD of sanitation and resource recovery systems. The implications of the deployment location on costs and life cycle GHG emissions were characterized via a contextual analysis considering country-specific factors (including average wages, grid electricity price and makeup, and bodily waste characteristics) for China, India, Senegal, South Africa, and Uganda. The results from this study provide insight into opportunities to advance the sustainability of the NEWgenerator and, more broadly, NSS through targeted research, development, and deployment of treatment and resource recovery technologies.

## Methods

### NEWgenerator Overview and Process Model

**Figure 1:**
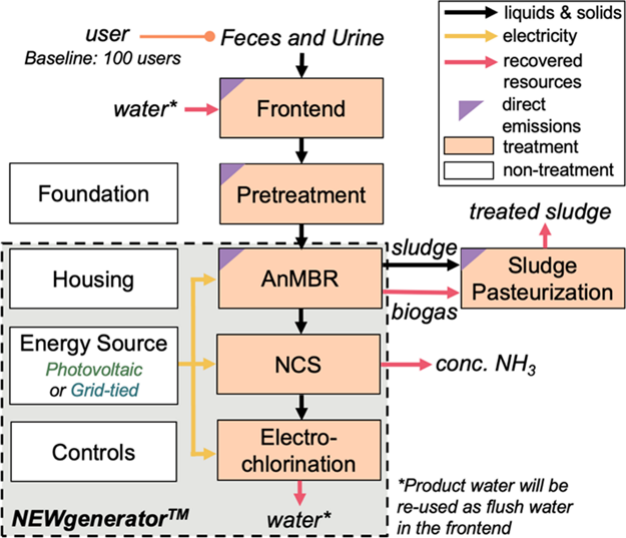
Process flow diagram summarizing the prefabricated NEWgenerator units (within the gray box with a black dashed border) and additional units included in this analysis (outside of the gray box). The color of the boxes represents ancillary units (white) and treatment processes included both in the design of the NEWgenerator and the onsite pre-treatment (orange). The boxes with purple triangles on the upper left corner represent unit processes from which fugitive emissions (e.g., N_2_O) are released. There are two different energy configurations for the NEWgenerator: photovoltaic and grid-tied. The inputs to and outputs from the system and between units are categorized as liquids and solids (black arrows), electricity (yellow arrows), and recovered resources (red arrows). Outputs across the evaluated scenarios include four recovered resources: biogas from the anaerobic membrane bioreactor (AnMBR), treated sludge from the sludge pasteurization unit, liquid NH_3_–N from the ion exchange unit in the nutrient capture system (NCS), and water from the electrochlorination (EC) unit to be recycled for use as flush water in the frontend.

The NEWgenerator consists of three main treatment processes: (i) an AnMBR for COD and solid separation and breakdown accompanied by methane production, (ii) an NCS consisting of ion exchange (with zeolite) for nitrogen (and incidental phosphorus) removal and recovery and granular activated carbon (GAC) for COD polishing, and (iii) an EC system for disinfection (Figure 1). Other ancillary units include process controls, a mini-shipping container to house the full treatment system, and a photovoltaic power unit (including photovoltaic panels, battery, DC distribution box, wiring, and other smaller required components) or a grid-tied power unit (including AC conversion, DC distribution box, wiring, and other smaller required components) as the energy source. All treatment data used for this work were from a 534-day field trial of the NEWgenerator 100 v.2.0 from October 2018 to March 2020 at an informal settlement community in eThekwini Municipality, KwaZulu-Natal Province, South Africa.^8^ The system treated high-strength wastewater from a community ablution block (which operated as the frontend), where wastewater from roughly 100–200 users was fed into a valve chamber, a bar screen, and an underground equalization tank before entering the NEWgenerator system. Because the field trial was intended to test the limits of performance, identify modes of failure, and determine maintenance requirements of the NEWgenerator; the system was, at times, pushed into operational conditions that did not meet ISO requirements. Temporal data were analyzed to determine the percentage of time that the liquid effluent and residual solids from this trial period did meet ISO 30500 requirements,^8^ specifically COD concentration, TSS concentration, *Escherichia coli* maximum concentration and LRV, pH range, TN removal, and TP removal (Table 1). The two different usage categories for the effluent were assessed for COD, and TSS are the threshold for unrestricted urban usage (Category A) and the threshold for discharge into surface water or other restricted urban use (Category B). Additional details about the performance of the NEWgenerator system were reported by Shyu et al.^8^ and Castro et al.^21^

**Table 1:** ISO 30500 Requirements for Liquid Effluent and Solid Outputs from NSS Systems and the Percentage of Time that the NEWgenerator Met those Requirements Based on Temporal Data from the 534-Day South Africa Field Trial^a^

parameter^b^	ISO 30500 requirements [% of time NEWgenerator met requirements]	
COD	A: <50 mg/L [29%]	B: <150 mg/L [64%]
TSS	A: <10 mg/L [63%]	B: <30 mg/L [98%]
TN	70% minimum load reduction [77%]	
TP	80% minimum load reduction [2%]	
*E. coli*	>6 LRV [84%]	
pH	6–9 [98%]	

a COD and TSS compliance are dependent on effluent usage, with category A being requirements for unrestricted urban use and category B being requirements for discharge into surface water or other restricted urban use. It should be noted that the purpose of the field trial was to test the limits of the NEWgenerator’s performance capabilities and identify modes of failure, and improved performance and stability would likely be achieved in commercial deployment scenarios.

b COD, chemical oxygen demand; TSS, total suspended solids; TN, total nitrogen; TP, total phosphorus; LRV, log reduction values.

We modeled the construction and performance of the NEWgenerator using the QSDsan^20^ python package.^22^ QSDsan enables an integrated workflow of design, simulation, techno-economic analysis (TEA), and life cycle assessment (LCA) under uncertainty.^23^ The baseline NEWgenerator was designed to treat bodily waste from 100 users over a 25-year system lifetime. Since the NEWgenerator is a backend system only, the frontend, pretreatment, foundation, and sludge pasteurization units were also included to evaluate the complete NSS system’s costs and environmental impacts. The frontend was based on an existing frontend toilet unit in QSDsan, assuming 1 seated toilet and 1 urinal per 25 people (4 seated toilets and 4 urinals for 100 users). Wastewater collected from the frontend (containing feces, urine, flush water, toilet paper, etc.) will first enter the pretreatment unit for flow equalization and trash removal. The pretreatment unit was designed using the onsite specifications for the equalization tank and bar screen from the South Africa field trial (Section S2 of the Supporting Information [SI]).^8^ The wastewater is then treated by the AnMBR, where the liquid effluent is subsequently processed by the NCS and EC units, and the sludge from AnMBR will be processed by a pasteurization unit. The concrete foundation was sized to support all treatment units. Spatial treatment performance from the South Africa field trial was simulated in the process model using mean removal values for the AnMBR (COD: 82%; TN: 20%; TP: 19%), NCS (COD: 63%; TN: 79%; TP: 36%), and EC units (COD: 18%; TN: 22%; TP: 11%).^8^ Although the system in the South Africa field trial did not include a sludge treatment unit, a sludge pasteurization unit was modeled for onsite solid treatment to comply with ISO 30500 solids output requirements.^19^ This unit consisted of a hydronic heat exchanger system and pump from an Omni Processor^18^ and was assumed to service 10 NEWgenerators. The pasteurization process was designed to achieve 70 °C for 30 min,^24^ and energy needed for this process was provided by biogas from the AnMBR with liquefied petroleum gas (LPG) as a supplement. Input streams of the system include consumables such as zeolite, GAC, sodium chloride (NaCl), sodium hydroxide (NaOH) in the NCS unit, as well as LPG in the sludge pasteurization unit. Output streams include biogas from the AnMBR unit, treated sludge from the sludge pasteurization unit, and wasted zeolite, wasted GAC, and concentrated liquid NH_3_–N from the NCS unit. TEA and LCA of the NEWgenerator were performed considering the system’s entire lifetime to quantify per capita daily costs and annual GHG emissions from capital, operation and maintenance (O&M; e.g., replacements, consumables), electricity, and direct sources (e.g., fugitive gaseous emissions from the treatment units). Two alternatives, photovoltaic power and grid-tied electricity, were considered as potential energy sources. The general case assumptions for cost include the capital and replacement components, materials, and consumables according to USA-build (used in South Africa field trial) and a global average grid electricity unit cost was used for the grid-tied configuration (Table S3). For assumptions related to GHG emissions, global average characterization factors were used for electricity GHG emissions, materials, components, and consumables (Table S4). Additional details on system configuration and analyses can be found on GitHub.^25^

### Techno-Economic Analysis

We performed TEA^26^ of the full NSS system to determine per capita costs using discounted cash flow analysis. For the general case, the capital costs for all components, units, and materials were estimated using the bill of materials (BOM) from the construction of the NEWgenerator in Tampa, Florida (USA). For the contextual analysis, we estimated capital costs across locations using country-specific price level ratios for BOM components which can potentially be manufactured or purchased locally (Table S5).^27^ Capital costs of NEWgenerator components were scaled using a learning curve and a production volume of 100,000 units (adapted from ref (18); Table S2, Section S3 of the Supporting Information).^28−30^ The deployment of 100,000 units could help meet the needs of 10+ million users, representing <0.3% of the global sanitation gap for safely managed services.^4^ We assumed that shipping costs associated with transportation of raw materials were embedded in the material price, and shipping costs for prefabricated components (for large-scale deployment) could be minimized (to be a small fraction of capital costs) with large-scale deployment (∼100,000 units) of the technology. O&M costs consisted of component replacements, consumables, energy (i.e., electricity and LPG), and labor expenses incurred over the lifetime of the system. We excluded the cost of land from the TEA due to its high variability and sensitivity to contextual factors, including the potential for the site to be publicly owned. Component replacement costs were annualized according to their respective lifetime if it was less than the system’s 25-year lifetime. The replaced component annual cost for useful life beyond the system lifetime were not considered in the analysis. Electricity requirements for the grid-tied configuration were based on the reported energy consumption from the Tampa, Florida (USA) integration testing (tested by the design team in 2018 using a variable power supply set at 24 V). Labor expenses were based on the detailed maintenance and replacement labor time requirements provided by the design team as well as labor wage prices.^31^ Finally, we applied a discount rate of 5% to determine the final cost per capita per day in United States Dollars (USD cap^–1^ day^–1^). Additional details related to the TEA are provided in Sections S1–S3 of the Supporting Information and in the QSDsan code.^32^

### Life Cycle Assessment

To characterize the life cycle GHG emissions of the NSS system, we performed an LCA across the construction and operational stages. The goal of the LCA was to elucidate key drivers of GHG emissions and to quantify the implications of targeted improvements to the NEWgenerator system. All sources of GHG emissions were normalized to CO_2_-equiv (100-year time horizon) with a functional unit of the provision of sanitation for one person for one year (final unit of kg CO_2_-equiv cap^–1^ year^–1^). The system boundary for this study included the construction, operation, and maintenance of the frontend and pretreatment units, the NEWgenerator, the system foundation, and sludge pasteurization unit (i.e., all units shown in Figure 1) over a 25-year lifetime. Material requirements for construction and replacement parts were acquired from the BOM, and consumables and energy consumption were determined from Tampa, Florida (USA) integration testing data. Life cycle inventory data for all materials and processes were acquired from the ecoinvent v3.2 database, and characterization factors were from the U.S. EPA’s Tool for the Reduction and Assessment of Chemicals and Other Environmental Impacts (TRACI 2.1 v1.03).^33,34^ For the general and country-specific cases, rest of world or global emission factors were used (when available) to account for global manufacturing and deployment. When material masses were not available in the BOM, masses were calculated using material density and volume or by identifying surrogates (similar items with masses available). Direct emissions (fugitive methane, CH_4_; nitrous oxide, N_2_O) from the degradation of bodily waste during storage and treatment were estimated using COD and nitrogen quantities in the bodily waste.^35−39^ A 90% biogas combustion efficiency was assumed for the sludge pasteurization unit (i.e., 10% of methane was released as a fugitive emission), and a 10% heat loss was assumed in the sludge heating process.^40^ The final effluent from the NSS system has the potential to be recycled for use as flush water but would require Category A treatment per ISO 30500.^19^ Ultimately, however, local regulations and conditions are likely to govern the reuse of recycled water. The transportation of construction materials and consumables, the disposal of the NEWgenerator at end of its life, and any off-site treatment and disposal of wastewater and solids were considered to be outside the scope of this analysis. Additional details of the LCA methodology are provided in Section S6 of the Supporting Information.

### Uncertainty and Sensitivity Analyses

To characterize the uncertainty in TEA and LCA results, probability distributions were defined for 154 input parameters for both photovoltaic and grid-tied configurations and used in Monte Carlo simulations. Latin Hypercube Sampling^41^ was used to generate 10,000 sets of input parameters for each configuration and improvement scenarios. Input values for parameters were determined from the BOM, vendors, manufacturers, and literature; distributions of these uncertain parameters were determined based on the quality and consistency of data. We used more constrained uncertainty (+/–15%) for values provided directly from the BOM, and less constrained uncertainty (25–35%) for costs and weights that were calculated, found externally on vendor or manufacturer websites, or when surrogates were used (e.g., tank specifications). A +/–20% uncertainty was applied to energy and labor requirements provided by the design team. Uniform distributions were used for all uncertainty ranges except when values were available from the literature and data supported a triangular distribution. To better understand the relative sensitivity of TEA and LCA results to each uncertain input parameter, Spearman’s rank-order correlation coefficients were calculated. Additional details are provided in Section S7 of the Supporting Information.

### Contextual Analysis

A contextual analysis was performed to account for location-specific parameter impacts on costs and environmental sustainability in comparison with the general case.^18,20^ The countries included in this analysis were China, India, Senegal, South Africa, and Uganda, of which the NEWgenerator has been previously piloted in India and South Africa.^8,42^ The countries were selected given readily available contextual data and to align with past studies that evaluated the deployment of complementary NSS technologies.^18^ Location-specific parameters included the unit grid electricity cost,^43^ the GHG intensity of local grid electricity,^44^ price level ratio,^27^ tax rate,^45^ labor wages,^31^ local diets (consumption of vegetal protein, animal protein, total caloric intake),^46^ food waste ratio,^47^ LPG price,^48^ and NaCl price (Table S5). The location-specific price level ratio was applied to the general case BOM costs to account for differing costs of materials, consumables, and components if sourced locally.

The location-specific trade-off in relative costs and life cycle GHG emissions between photovoltaic and grid-tied power was driven by the unit cost and GHG intensity of the local grid electricity. Thus, the NEWgenerator was simulated across a wide range of unit grid electricity costs (0.0–0.6 USD kWh^–1^) and grid GHG emissions (0–1 kg CO_2_equiv kWh^–1^). These ranges capture the lowest to highest unit grid electricity costs^43^ and emissions^44^ based on datasets from across the globe and can inform discussion about the implications of local electricity sources on the relative sustainability of the photovoltaic versus grid-tied NEWgenerator. Rest of the world or global emission factors were used across the contextual analysis for consistency due to data insufficiencies regarding location-specific manufacturing of components and consumables (e.g., photovoltaic panels, NaCl).

### Simulation of Targeted Improvements

To characterize the cost and GHG implications of targeted improvements to the NEWgenerator, five potential improvement scenarios were evaluated: (Scenario 1) increase system capacity from 100 (baseline) up to 600 users; (Scenario 2) develop or source lower cost or lower impact components and services, including the use of a lithium photovoltaic battery, sourcing of a lower cost housing, more efficient sludge pasteurization service, and use of a frontend with reduced O&M requirements; (Scenario 3) reduced O&M requirements for NCS by replacing zeolite instead of regenerating it and increasing the zeolite capacity for NCS; (Scenario 4) dissolved methane recovery via hollow fiber membrane contactor (HFMC) installed post-AnMBR to reduce direct GHG emissions; and (Scenario 5) concentration and sale of recovered NH_3_ as a liquid fertilizer. Notably, although the NEWgenerator has been designed for 100 users, it has been tested with an estimated 100–200 users in the South Africa field trial. Furthermore, we simulated underutilization (down to 50 users; results in the Supporting Information) and hypothetical increases in system loading in Scenario 1 to characterize the cost and GHG implications of these use cases. Additionally, nutrient recovery, sales, and emission offsets were not considered in the baseline scenario as the current design would require additional treatment units to achieve commercial quality nutrients. However, the potential of recovered nutrients to reduce cost and GHG emissions was evaluated in Scenario 5.

## Results and Discussion

### Financial Viability and Environmental Performance of the NEWgenerator

**Figure 2:**
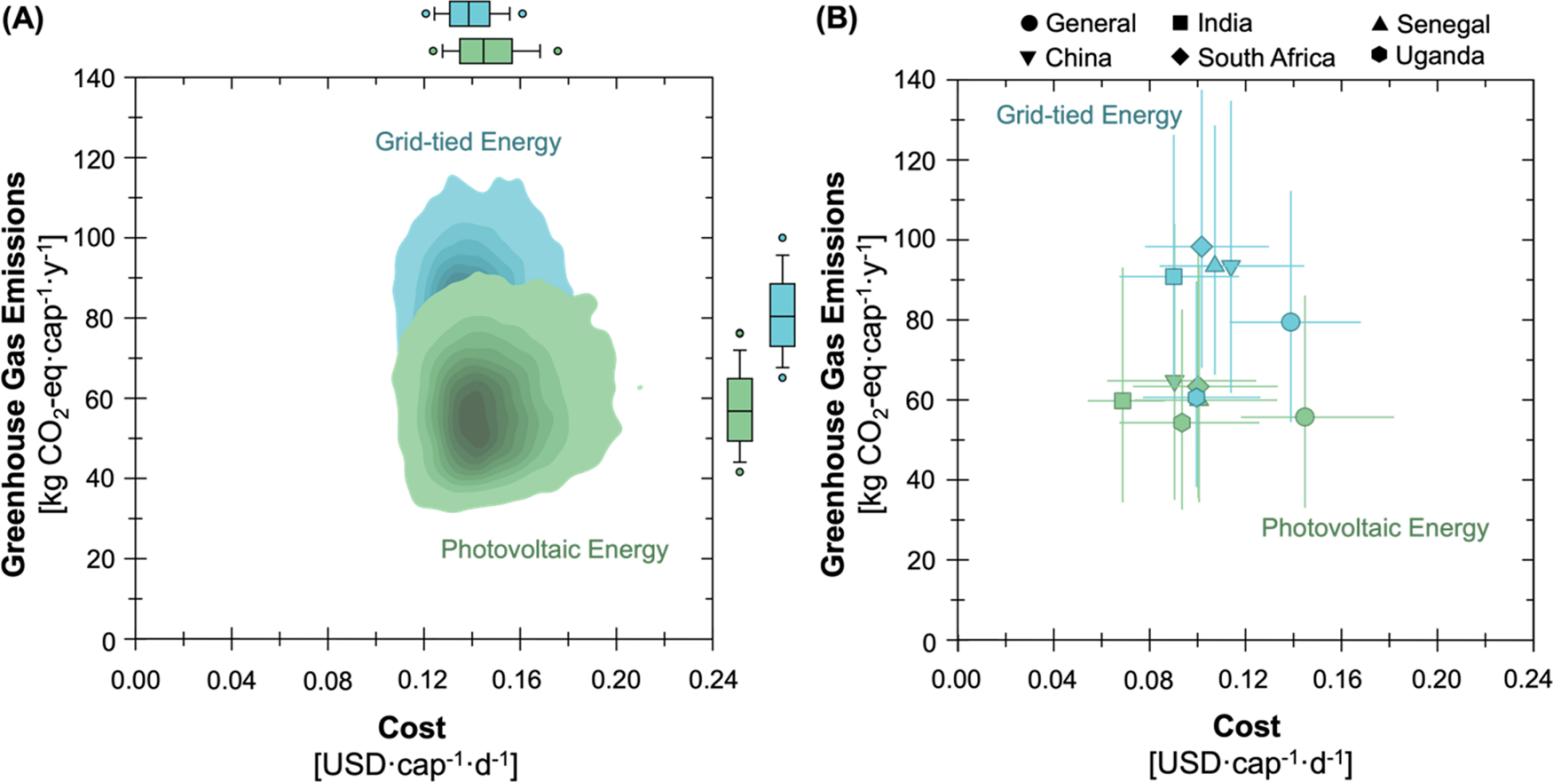
Estimates of economic and environmental outcomes associated with the NEWgenerator under (A) two different energy configurations (photovoltaic, green; grid-tied, blue) and (B) different deployment contexts (the general case and five countries of interest). Shading in the kernel density maps in (A) represents the density of results from 10,000 Monte Carlo simulations (darker regions have a higher density of results). The horizontal position corresponds to per capita cost and the vertical position corresponds to per capita GHG emissions. Box and whisker plots represent the same data as the kernel density maps and show median, 25th/75th, 10th/90th, and 5th/95th percentiles with the center line, bottom/top of the box, lower/upper whiskers, and points below/above the whiskers, respectively. In (B), the points represent the baseline values and the error bars represent 5th/95th percentiles from 10,000 Monte Carlo simulations, and the color scheme is consistent with (A).

The economics and environmental impacts of the baseline NEWgenerator were characterized under general case assumptions (Figure 2A) and across five countries of interest (Figure 2B). Under the general case assumptions, the use of photovoltaics for on-site electricity production resulted in lower GHG emissions but at a higher per capita cost when compared to grid-tied electricity (Figure 2A). The photovoltaic energy configuration median per capital cost was 0.145 USD cap^–1^ day^–1^, with a 5th and 95th percentile range of 0.118–0.181 USD cap^–1^ day^–1^ [hereafter, the 5th and 95th percentile range will be shown in brackets following the median value]. The grid-tied energy configuration was approximately 4% cheaper compared to the photovoltaic configuration, at 0.139 [0.113–0.168] USD cap^–1^ day^–1^. The per capita GHG emissions when using photovoltaics were 56.1 [33.8–86.2] kg CO_2_-equiv cap^–1^ year^–1^, which was 30% lower than the grid-tied configuration at 79.7 [55.0–112.3] kg CO_2_-equiv cap^–1^ year^–1^. The total lifetime cost for a single NEWgenerator was 132,000 [108,000–165,000] USD and 127,000 [103,000–153,000] USD for photovoltaic and grid-tied configurations, respectively. The total lifetime GHG emissions for the system were 140,000 [85,000–216,000] kg CO_2_-equiv and 199,000 [138,000–281,000] kg CO_2_-equiv for photovoltaic and grid-tied configurations, respectively. The increase in per capita cost for the photovoltaic configuration (as compared to grid-tied) for the general case can be attributed to the capital and replacement costs of the photovoltaic panels and battery bank: these components incurred greater costs than grid-tied electrical components and the consumptive cost of grid electricity over the 25-year lifetime. The grid-electricity consumption of treatment units accounted for approximately 23.4 [21.0–25.8] kg CO_2_-equiv cap^–1^ year^–1^ of GHG emissions or 29 [26–32]%. Ultimately, the decision to reduce GHG emissions by transitioning from grid electricity to solar-powered is an expensive way to mitigate GHGs in the general case (at a grid electricity unit cost of 0.06 USD kWh^–1^), incurring 91.1 [83.1–192.7] USD Mg CO_2_-equiv^–1^ in the general case scenario as compared to (for example) proposed carbon pricing of 34–64 USD Mg CO_2_-equiv^–1^ in the United States in 2025 to achieve net-zero CO_2_ emissions by 2050.^49^ Instead, the selection of a photovoltaic system may be driven more by lack of access to reliable grid-based electricity (e.g., in rural settings or locations with unreliable electric grids).

In comparison to other sanitation systems, both photovoltaic and grid-tied configurations of the NEWgenerator had higher costs than pit latrines integrated with centralized anaerobic treatment (0.02 to 0.07 USD cap^–1^ day^–1^),^18,50^ but comparable or lower GHG emissions (estimated ranges include 23–86^50^ and 85–115^18^ kg CO_2_-equiv cap^–1^ year^–1^). In relation to other NSS treatment technologies, costs were inversely correlated with the system size: the NEWgenerator incurred lower costs than an NSS liquid treatment technology serving six users (0.19 [0.13–0.26] USD cap^–1^ day^–1^)^51^ but higher costs than an Omni Processor technology serving 12,000 users (0.05 [0.03–0.09] USD cap^–1^ day^–1^ with pit latrines as the frontend).^18^ It is important to note that there are minor methodological differences across studies (e.g., TEA parameters), and direct comparisons would require future work that evaluates NSS alternatives with consistent contextual assumptions.^23^

While the cost and GHG emissions of the general case provided an initial estimate of the relative sustainability of the NEWgenerator using a global average price ratio for BOM costs and global average emissions factors, understanding how contextual factors impact the economics and environmental impact of the NEWgenerator is necessary to inform intentional deployment decisions. All five countries had lower per capita costs than the general case because (i) the price level ratios for the five countries were less than 1.0 (the reference price level ratio of the U.S., 1.0, was used in the general case) and (ii) the country-specific consumables and grid-electricity prices are lower. Grid-tied configurations had lower costs but higher emissions than photovoltaic configurations in India, China, and South Africa, whereas assumptions of grid characteristics in Senegal and Uganda resulted in lower costs and emissions for the photovoltaic configuration (Figure 2B). The daily cost of the grid-tied and photovoltaic configurations was below 0.114 and 0.100 USD cap^–1^ day^–1^ across all five countries, respectively. The estimated cost in India was the lowest of the five countries with median values of 0.090 and 0.069 USD cap^–1^ day^–1^ for grid-tied and photovoltaic configurations, respectively, followed by Uganda (0.099 and 0.093 USD cap^–1^ day^–1^), South Africa (0.102 and 0.100 USD cap^–1^ day^–1^), Senegal (0.107 and 0.100 USD cap^–1^ day^–1^), and China (0.114 and 0.090 USD cap^–1^ day^–1^).

Most countries had higher GHG emissions than the general case, except for Uganda which was 1.4 and 18.7 kg CO_2_equiv cap^–1^ year^–1^ lower for photovoltaic and grid-tied sources, respectively. The grid-tied configurations across countries resulted in 28.6–34.8 kg CO_2_-equiv cap^–1^ year^–1^ higher GHG emissions than their photovoltaic counterparts, except in Uganda which was only 6.3 kg CO_2_-equiv cap^–1^ year^–1^ (Figure 2B). Observations unique to Uganda stemmed from the country’s primary source of electricity, hydropower, which had a low GHG intensity but high unit cost (second in cost only to Senegal; see Tables S4 and S5 for details about grid electricity assumptions across countries). Due to increased reliance on coal and oil for electricity production (relative to Uganda), China, India, Senegal, and South Africa had higher median GHG emissions for grid-tied configurations ranging from 91.1–98.6 kg CO_2_equiv cap^–1^ year^–1^, while Uganda had significantly lower GHG emissions at 61.1 kg CO_2_equiv cap^–1^ year^–1^.

Beyond unit costs and GHG intensity of grid electricity across countries, assumptions about diet (consumption of vegetal protein, animal protein, total caloric intake) and caloric intake also influenced the performance of both configurations due to changes in influent COD and nutrients and subsequent changes in O&M (Figures S2 and S3). Given the differences in costs and GHG emissions between the general case and country-specific scenarios (Figure 2B), it is clear that contextual parameters can influence the economic and environmental sustainability of the NEWgenerator. Locality-specific manufacturing assumptions could be revisited in future work to support more in-depth, community-specific evaluations. For example, if it is known that the photovoltaic panels will be sourced from China (which accounts for 62% of total photovoltaic panel production worldwide^52^), global average emissions factors could be replaced with values representative of typical production practices in China. Similarly, community-specific diets, material pricing, and consumable pricing (and their associated uncertainties) can be considered when known. Ultimately, the inclusion of more detailed contextual parameters can provide more accurate results in a given deployment context and should be pursued when performing community-specific evaluations.

### Elucidating Key Drivers of Cost and GHG Emissions

**Figure 3:**
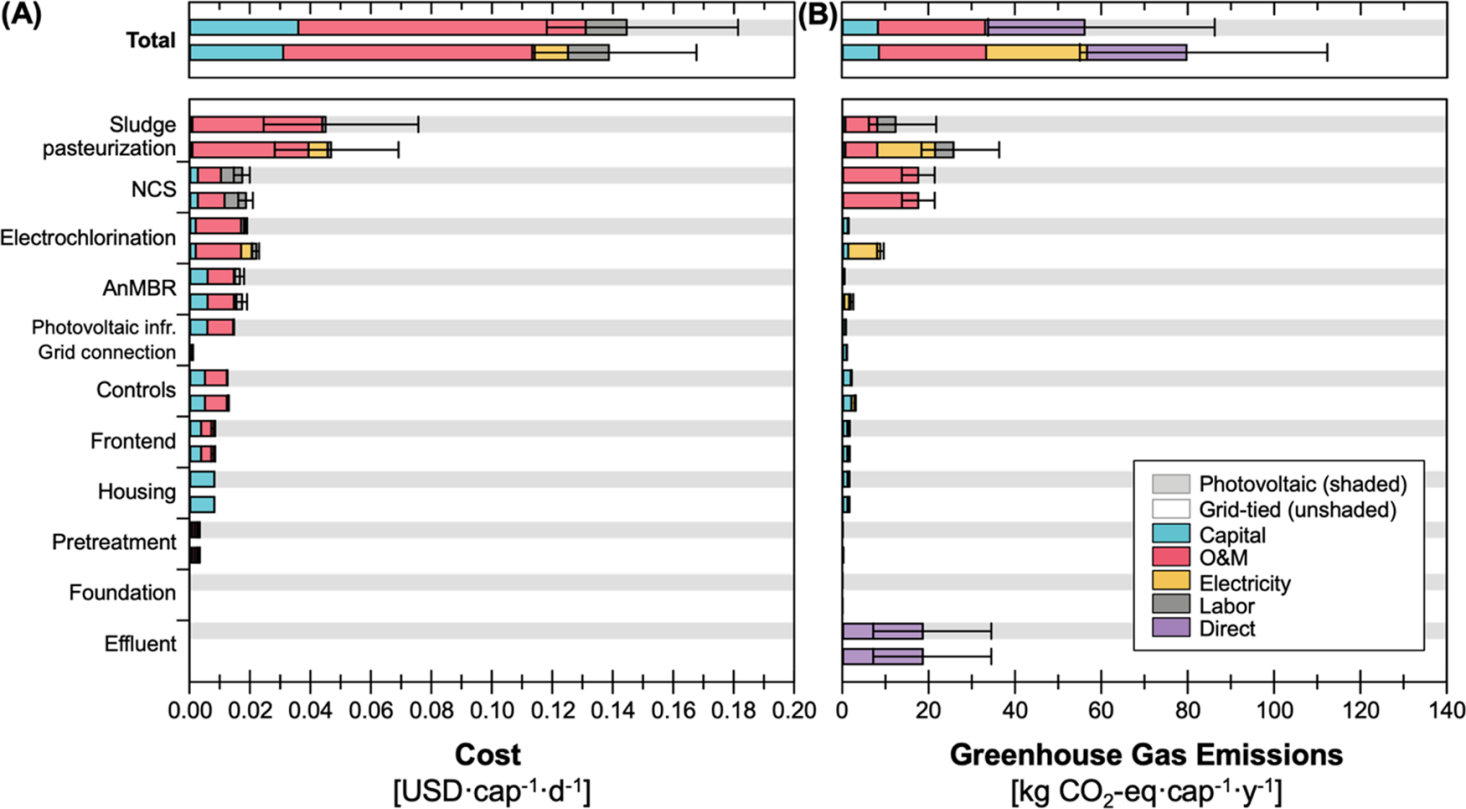
(A) Daily per capita cost estimates and (B) annual per capita life cycle GHG emissions for the NEWgenerator technology assuming 100 users, a 25-year lifetime, and 100,000 units produced at scale for two different energy configurations (photovoltaic and grid-tied). The shaded background bars depict the photovoltaic configuration (gray), and the unshaded background depicts the grid-tied configuration. The top panel shows the total breakdown of costs and GHG emissions by relative contributions from capital, O&M, electricity, labor, and direct (for GHG emissions only) to the median per capita cost. The lower panels further break down per capita cost and GHG emissions by each unit process. Error bars extend to the 5th and 95th percentile values from the uncertainty analysis.

The key drivers of per capita cost and GHG emissions were identified by characterizing the contributions from each unit (Figure 3). For both energy source configurations, the per capita cost was driven by the replacement costs (captured within O&M) of components with shorter lifetimes than the system lifetime (e.g., 2–10 year pump lifetimes, 2-year electrochlorinator lifetime), labor costs associated with O&M, capital costs of the key components (e.g., pumps, reactors), and consumables used in treatment (e.g., zeolite, GAC, NaCl, and NaOH in NCS, LPG in sludge pasteurization). For all units except the NCS and Pretreatment, the O&M and capital were the first and second largest contributors, respectively, to per capita cost. The second largest cost for the NCS and Pretreatment was labor, which stemmed from the labor requirements for zeolite regeneration and screen cleaning, respectively, and pump replacements for both units. The median relative costs across unit operations for the photovoltaic configuration were the highest for the sludge pasteurization (31%), the NCS (12%), EC (13%), and AnMBR (12%) treatment units, followed by the power unit (10%) and controls (9%; Figure 3). The relative costs across unit operations for the grid-tied configuration were similar, with the highest contribution for the sludge pasteurization (34%), NCS (13%), EC (16%), and AnMBR (13%) treatment units, followed by controls (9%) and the power unit (1%; Figure 3). The cost contribution of grid-tied power consumption was minimal to the overall cost (8%), and the grid-tied unit had lower capital and O&M costs than the photovoltaic unit, the latter of which included photovoltaic panels and a battery.

The relative GHG contributions varied across units, but trends were relatively consistent between photovoltaic and grid-tied configurations. For the photovoltaic configuration, the median contributions to life cycle GHG emissions were the highest for fugitive emissions from effluent (33%), followed by NCS (31%), sludge pasteurization (22%), and EC (2%; Figure 3). For the grid-tied configuration, median contributions to life cycle GHG emissions were the highest for fugitive emissions from effluent (23%) followed by sludge pasteurization (32%), NCS (22%), and EC (11%; Figure 3). Direct emissions can be attributed to fugitive methane leaving the system as dissolved methane in the effluent and residual methane from biogas combustion in the sludge pasteurization unit. The O&M emissions are primarily attributed to the production of the consumables in the sludge pasteurization (specifically, LPG) and the chemically driven NCS units (including zeolite, GAC, NaCl, and NaOH). In the grid-tied configuration, the emissions from electricity consumption are predominantly from the sludge pasteurization unit (13.4 [12.2–14.6] kg CO_2_-equiv cap^–1^ year^–1^) and EC unit (7.5 [6.8–8.1] kg CO_2_-equiv cap^–1^ year^–1^). When low GHG energy sources are used (including with the photovoltaic configuration), there are tangible emissions benefits from selecting electrically driven treatment processes in NSS technologies. Beyond disinfection, the electrification of nutrient removal also has the potential to reduce costs and GHG emissions. It is also important to note that the foundation, frontend, pretreatment, and foundation had negligible impact on the overall costs and emissions of the NEWgenerator.

Sensitivity analysis was used to quantify the Spearman’s rank-order correlation coefficient of assumptions and parameters in the analysis to determine which had the greatest influence on the uncertainty in cost and GHG emissions of the NEWgenerator (Figures S2 and S3). The per capita cost was highly sensitive to sludge moisture content with a Spearman’s rank-order correlation coefficient of 0.58 for both the photovoltaic and grid-tied configurations. This was followed by urine excretion (0.37 and 0.36 for the photovoltaic and grid-tied configurations, respectively), fecal excretion (0.38 for both configurations), LPG price (0.33 for both configurations), and labor wages (0.15 and 0.14; Figure S2). Although sludge moisture content was a key driver of cost uncertainty as it directly relates to the amount of heat (and therefore volume and cost of fuel) needed to dry and pasteurize the sludge from its native water content, it was not one of the key drivers of life cycle GHG emissions (Figure S3). The per capita GHG emissions were most sensitive to the users’ energy excretion (fraction of intake; coefficient of 0.91 for both configurations), which influences the amount of COD in the waste stream and ultimately how much methane is produced (and released as a fugitive emission). This was followed by the Spearman’s rank-order correlation coefficient of AnMBR methane yield/production fraction (0.22), AnMBR COD removal (0.19), caloric intake (0.17), and methane characterization factor (0.17; the Spearman’s rank order correlation coefficients for life cycle GHG emissions were consistent across the photovoltaic and grid-tied configurations). All the above parameters important to GHG emissions directly relate to the influent COD and its degradation to methane as well as the sludge characteristics which correspond to the amount of fuel needed for sludge pasteurization.

### Targeted Improvements to Advance Sustainability

#### Increase User Capacity

**Figure 4:**
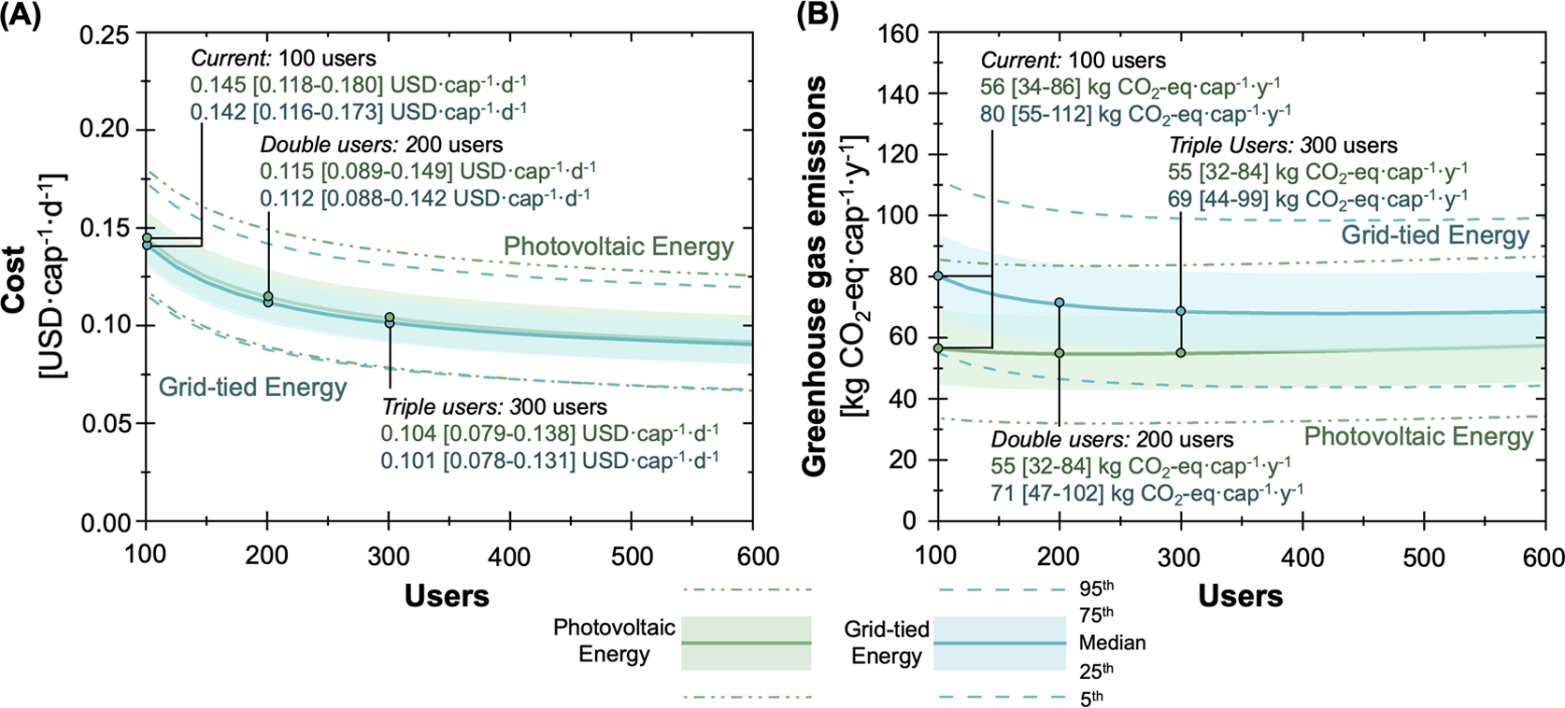
(A) Daily per capita cost and (B) annual per capita GHG emissions were simulated based on the impact of increasing users (increasing hydraulic throughput and COD/nutrient loading rates) for the NEWgenerator general case. Two different energy configurations were simulated: (green, top values in annotation) photovoltaic and (blue, bottom values in annotation) grid-tied. The user capacity was simulated from 100 users (baseline or current) to 600 users, with per capita cost and emissions for current, double, and triple users highlighted with annotation. The value of 600 users was selected as the maximum value for the *x*-axes to clearly show diminishing financial benefits at higher user loadings. The median, 25th/75th, and 5th/95th are depicted by the solid line, shaded region, and dashed line, respectively, to represent the range of results from uncertainty analysis. Simulation results from the underutilization analysis (50 actual users with a system sized for 100 users) are provided in Figure S1 of the Supporting Information.

Increasing the loading rate (hydraulic, COD, and nutrient loading) to the NEWgenerator was modeled by changing the number of users and the corresponding influent flowrate. The South Africa field trial demonstrated robust treatment performance of high strength black and yellow water at an estimated 100–200 users.^8^ Thus, increasing the user capacity may be plausible for the NEWgenerator and was simulated to determine the cost and environmental implications. For this analysis, the per capita cost and life cycle GHG emissions were determined for scenarios of underutilization (50 users; Figure S1) up through increased loading to 300+ users (Figure 4A,B). The increased number of users was simulated using the NEWgenerator general case at a baseline design of 100 users, assuming core unit components (system housing, membranes, bioreactor, NCS and GAC tanks, etc.) could handle increased loading without adjustment. Additional components (pumps, the electrochlorinator, biogas storage, power systems, etc.), consumables (including zeolite, GAC, NaCl, NaOH), electricity demand, and maintenance requirements (including labor and component replacements) were scaled according to the number of users (Tables S7 and S8 in the Supporting Information).

Increasing the number of users reduced the per capita cost significantly (Figure 4A) but had limited impact on GHG emissions (Figure 4B). Doubling the number of users (from the baseline of 100 users) to 200 users reduces the median daily cost by 3.0 cents (from 0.145 USD cap^–1^ day^–1^ to 0.115 USD cap^–1^ day^–1^) for the photovoltaic energy configuration and 3.0 cents (from 0.142 USD cap^–1^ day^–1^ to 0.112 USD cap^–1^ day^–1^) for the grid-tied energy configuration. Continuing to increase the number of users had diminishing reduction in cost, with a plateau in photovoltaic and grid-tied median costs of approximately 9.2 and 9.0 cents per capita per day, respectively. O&M, electricity, and direct emissions were the main sources of the NEWgenerator GHG emissions (Figure 3) and scaled linearly as users increased. Thus, increasing the number of users had a negligible impact on GHG emissions per capita. If the NEWgenerator is sized for 100 users but underutilized at 50 users (half of its design capacity), the photovoltaic configuration median per capita cost and GHG emissions approximately doubled to 0.246 USD cap^–1^ day^–1^ and 82.3 kg CO_2_equiv cap^–1^ year^–1^, respectively (Figure S1). Ultimately, pushing the existing NEWgenerator design to the highest possible loadings (without significantly compromising treatment performance) may be an effective way to reduce costs and impacts in the near term.

#### Alternative Components and More Efficient Frontend/Residual Services

**Figure 5:**
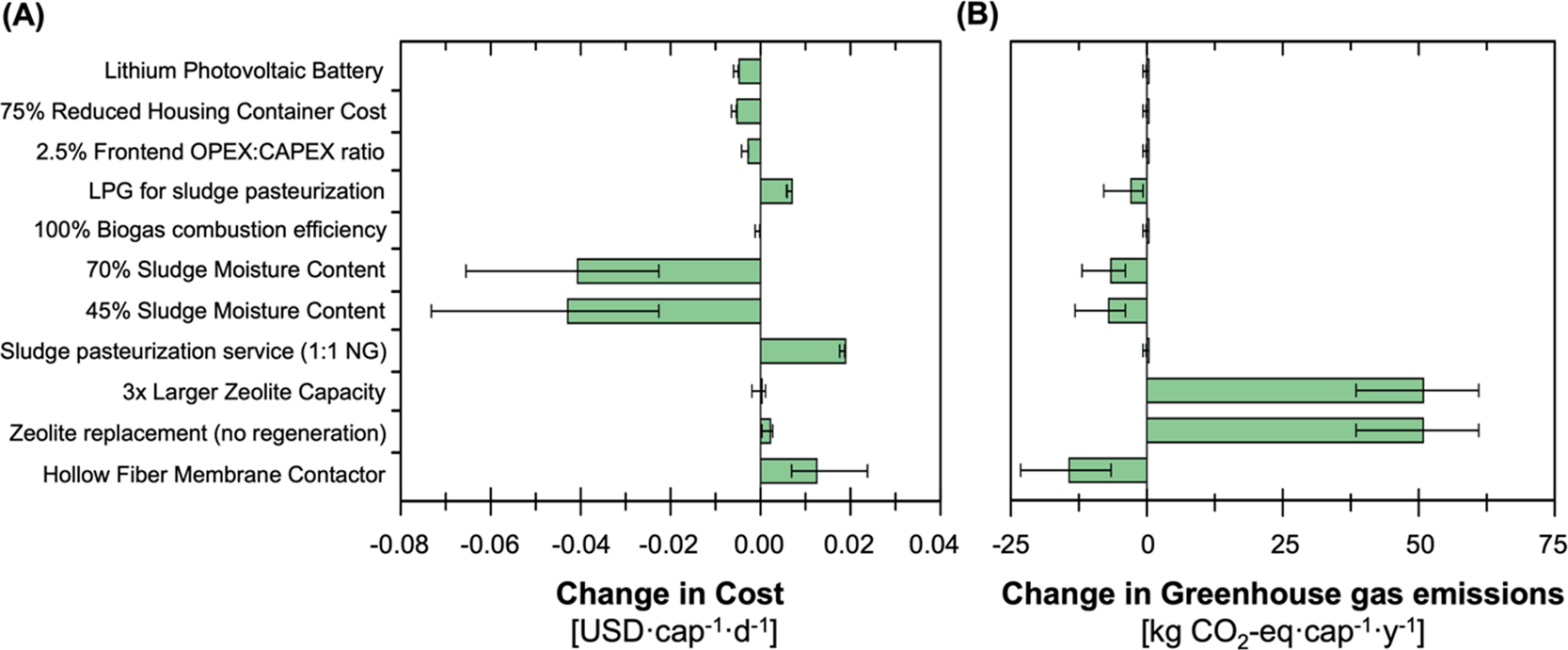
(A) Median change in daily per capita cost and (B) annual per capita GHG emissions of the NEWgenerator photovoltaic configuration general case from the baseline case were simulated for each targeted improvement to advance sustainability for the NEWgenerator. Error bars extend to the 5th and 95th percentile values from the uncertainty analysis.

Improvements to capital and replacement components using alternative components and more efficient frontend and residuals management services were simulated in this scenario. The alternative components included using a lithium photovoltaic battery with a longer lifetime (20-year compared to 11-year) and a 75% lower-cost standard shipping container for the housing. Assumptions related to increased efficiency of supporting operations assumed a reduction in frontend annual operating expenses from 7.5 to 2.5% of total capital expenses. Assumptions related to the sludge pasteurization service, such as lower sludge moisture content, higher biogas combustion efficiency, LPG as the fuel source for pasteurization, and single sludge pasteurization unit for one NEWgenerator. The component changes (lithium photovoltaic battery and low-cost housing) each reduced median per capita cost by approximately 0.5 cents, while frontend 2.5% annual O&M (as a percent of total capital costs) decreased it by 0.3 cents; all had no substantive impact on GHG emissions (Figure 5A,B). Typical sludge moisture contents from additional drying (e.g., mechanical processes, drying beds, etc. without additional equipment accounted for)^53,54^ prior to the pasteurization unit was considered to reduce the moisture content from 93 [90–95%]^55^ and decrease consumable consumption in the unit. A sludge moisture content of 70 and 45%^53,54^ reduced median per capita cost by 4.1 and 4.3 cents, respectively, and GHG emissions by 6.6 and 7 kg CO_2_equiv cap^–1^ year^–1^, respectively (Figure 5A,B). Use of LPG-only for combustion decreased emissions by 2.9 kg CO_2_equiv cap^–1^ year^–1^ at an increased cost of 0.7 cents, while increasing biogas combustion efficiency to 100% had negligible impacts (Figure 5A,B). A 1:1 ratio of the sludge pasteurization unit to NEWgenerator would increase cost and GHG emissions by 1.8 cents and 0.4 kg CO_2_equiv cap^–1^ year^–1^, respectively (Figure 5A,B). If all the above positive improvements (component changes, 45% sludge moisture content, and 2.5% frontend annual O&M as a percent of total capital costs) are applied assuming no negative interactions among them, there is potential for median daily per capita cost and GHG emissions to be reduced by 39% to 0.089 USD cap^–1^ day^–1^ and 11% to 50.2 kg CO_2_equiv cap^–1^ year^–1^, respectively.

#### Alternative Zeolite Configurations in Ion Exchange

One of the key drivers of cost and GHG emissions is the ion exchange unit within the NCS which uses zeolite to remove nitrogen. The baseline design consists of 3 zeolite regeneration events per year and complete replacement every 3 years.^21^ Two alternative design scenarios for the NCS unit were evaluated, including (i) a zeolite *replacement only* scenario where zeolite is not regenerated and is instead replaced annually, and (ii) an *increased zeolite capacity* scenario where the zeolite capacity is increased 3-fold and replaced annually. The zeolite replacement only scenario and increased zeolite capacity scenario had negligible impact on per capita cost, while GHG emissions increased by 50.8 kg CO_2_equiv cap^–1^ year^–1^ for both scenarios (Figure 5A,B). Although the cost and emissions associated with regeneration labor and consumed NaCl (in regenerant solution) is reduced in both scenarios, the increased consumption of zeolite (from annual replacement) incurred equal costs and greater emissions. Therefore, the baseline design using zeolite regeneration would be preferable over these alternative zeolite configuration scenarios as reductions in labor and regeneration consumables do not outweigh the impacts of additional zeolite consumption.

#### Recovering Dissolved Methane Using a Hollow Fiber Membrane Contactor

A significant fraction of methane produced in AnMBRs generally remains dissolved in the liquid effluent, with reported values of 30–50% for AnMBR systems treating municipal wastewaters.^56,57^ For the NEWgenerator, if dissolved methane is released as a fugitive emission, it represents roughly 18.6 kg CO_2_equiv cap^–1^ year^–1^ (33%) of the total photovoltaic configuration GHG emissions (Figure 3). To reduce the dissolved methane leaving the system, a scenario involving the installation of a post-AnMBR HFMC was simulated. The micro-porous HFMC was selected for high liquid–gas separation (dissolved gas will diffuse through the membrane pores) and potential to maintain high fluxes and observe limited scaling.^58,59^ For this scenario, the HFMC was assumed to remove 63.3–98.9% (uniform uncertainty range) of dissolved methane based on process boundary conditions for separation which has been demonstrated for surrogate and real anaerobic effluents.^58,59^ The recovered dissolved methane was mixed with recovered biogas for sludge pasteurization. The addition of an HFMC comes at a tradeoff; a 14.3 kg CO_2_equiv cap^–1^ year^–1^ reduction in GHG emissions to a new system impact of 41.9 [27.2–63.0] kg CO_2_-equiv cap^–1^ year^–1^ is achieved at the expense of 0.013 USD cap^–1^ day^–1^ increase to 0.157 [0.125–0.205] USD cap^–1^ day^–1^ (a GHG mitigation cost of roughly 319 USD Mg^–1^ of CO_2_) (Figure 5A,B).

#### Resource Recovery To Offset Costs and Emissions

Resource recovery of the NH_3_–N recovered from the NCS unit via zeolite adsorption and desorption has the potential to further offset cost and emissions. At baseline operation, approximately 55.8 kg NH_3_–N year^–1^ is recovered in the liquid form in the wasted brine regenerant solution. Assuming that the NH_3_–N in that solution can be sold at market price for N fertilizer at 1.50 USD kg^–1^,^60−62^ the potential median cost offset would be approximately 0.0023 USD cap^–1^ day^–1^, which represented a minimal (2%) reduction on the per capita cost. Similarly, the median offset of GHG emissions would be 1.2 kg CO_2_equiv cap^–1^ year^–1^ or a 2% reduction from baseline GHG emissions assuming 2.16 kg CO_2_equiv kg NH_3_–N^–1^.^63^ To achieve this negligible benefit, however, further treatment of the liquid NH_3_–N in the regenerant solution would be required to reach market fertilizer quality, but the current design does not account for infrastructure and materials/energy required to achieve this additional purification step. Additionally, phosphorus recovered from the NEWgenerator can also serve as a fertilizer which could offset costs and emissions. The NEWgenerator has not met the ISO 30500^19^ treatment performance requirements for TP removal as the current design only passively removes TP, but future work that supports phosphorus recovery (e.g., via magnesium hydroxide addition) could achieve improved effluent quality and GHG/economic outcomes. One such direction could be to encourage phosphorus precipitation within the AnMBR unit or to require influent be source-separated (urine separated from feces, as in a urine diverting dry toilet) and to achieve precipitation in a urine holding tank.^60,64^

### Impact of Grid Electricity Characteristics on System Sustainability

**Figure 6:**
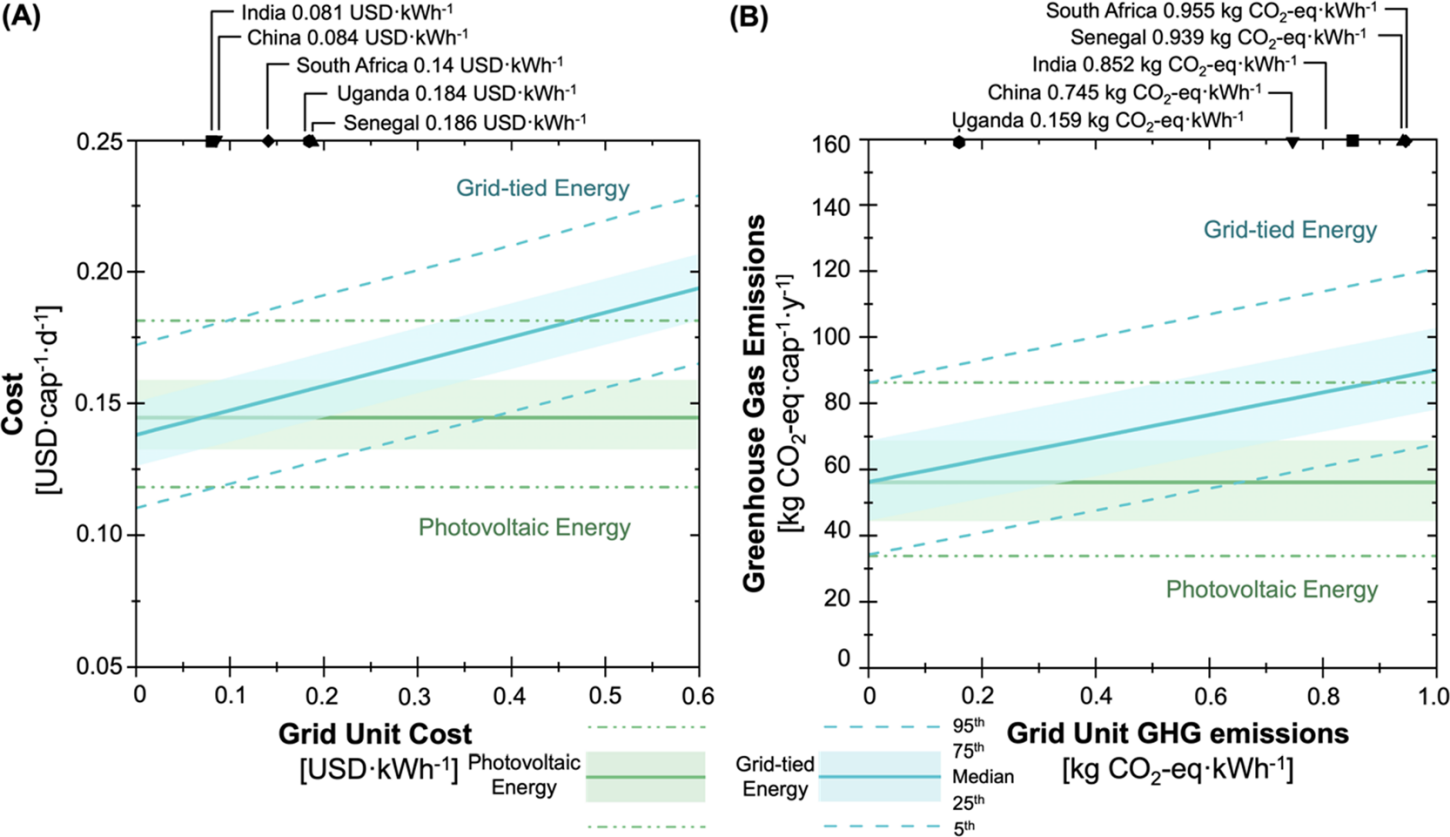
(A) Impact of grid unit electricity cost (from 0.00–0.60 USD kWh^–1^) and (B) grid electricity unit emissions (from 0–1 kg CO_2_equiv kWh^–1^) on the costs and impacts of the NEWgenerator. The (green) photovoltaic configuration per capita cost and life cycle GHG emissions are constant across all values, whereas the (blue) grid-tied configuration costs and impacts vary with grid characteristics. The country-specific values of grid electricity unit cost and unit emissions for the five countries of interest (China, India, Senegal, South Africa, and Uganda) are shown on the upper *x*-axes. The median, 25th/75th, and 5th/95th are depicted by the solid line, shaded region, and dashed line, respectively, to represent the range of results from uncertainty analysis.

To better understand how the unit costs and GHG emissions of local grid electricity impact the relative sustainability of the photovoltaic versus grid-tied energy configurations, the two systems were compared across potential grid characteristics (Figure 6A,B). A pair-wise comparison was conducted between the photovoltaic and grid-tied configurations across a range of grid electricity unit costs (0.00–0.60 USD kWh^–1^) and grid electricity unit emissions (0–1 kg CO_2_equiv kWh^–1^). The per capita cost of the grid-tied energy configuration was lower than the photovoltaic configuration when grid electricity unit costs fell below 0.07 USD kWh^–1^ (Figure 6A). Currently, the lowest and highest reported country-level household electricity price (0.003 USD kWh^–1^ in Lebanon and 0.37 USD kWh^–1^ in Bermuda^43^) are below and above this threshold. Although community-specific pricing may vary within these reported ranges, these results do suggest that the grid-tied configuration is likely to be less expensive than the photovoltaic configuration only at low unit electricity prices. The photovoltaic configuration has lower GHG emissions than the grid-tied configuration across nearly all possible unit GHG emissions for grid electricity (Figure 6B). The five countries (China, India, Senegal, South Africa, and Uganda) evaluated all have country-level household grid electricity prices above the 0.07 USD kWh^–1^ cost threshold (they ranged from 0.081–0.186 USD kWh^–1^) that resulted in minimal cost increase (1–7%) and, except for Uganda (0.159 kg CO_2_equiv kWh^–1^), all had grid GHG emissions that resulted in substantive (45–58%) GHG savings with the photovoltaic system. Therefore, when solely considering the location-specific grid electricity unit price and unit GHG emissions, all five countries have higher per capita costs and higher GHG emissions with the grid-tied configuration (contrary to the general case scenario results, which yielded a tradeoff between cost and life cycle GHG emissions because of a lower unit cost of electricity).

To navigate the tradeoffs between cost and GHG emissions in the choice between the photovoltaic or grid-tied energy configuration, the dollars per Mg of GHG emissions (USD Mg CO_2_equiv^–1^) were quantified to better understand the efficiency of using funds to mitigate GHG emission in this way. As a benchmark against which to compare, a near-term CO_2_ price in the United States to achieve a 2050 net-zero CO_2_ emission target is 34–64 USD Mg CO_2_^–1^ in 2025 and 77–124 USD Mg CO_2_^–1^ in 2030.^49^ For the NEWgenerator, the cost of mitigating life cycle CO_2_ emissions by selecting the photovoltaic configuration (instead of the grid-tied configuration) would be approximately −253 USD Mg CO_2_equiv^–1^ in India, −300 USD Mg CO_2_equiv^–1^ in China, −72 USD Mg CO_2_equiv^–1^ in Senegal, −15 USD Mg CO_2_equiv^–1^ in South Africa, and −348 USD Mg CO_2_equiv^–1^ in Uganda. The cost of mitigating carbon through configuration selection is not only below the benchmark proposed above (the 2025 and 2030 United States pricing) but also negative. This suggests that GHG mitigation from selection of the photovoltaic configuration will reduce the system cost and GHG emissions for any country with electricity price above 0.07 USD kWh^–1^. In addition to the adequate cost and environmental justification for this selection, there are intangible benefits for use of photovoltaic energy in locations which lack grid infrastructure or experience inconsistent electricity supply.

### Prioritizing Paths Forward for Technology Development and Deployment

This research assessed the financial viability and environmental implications of the NEWgenerator, a NSS system, and provided insight into the implications of targeted improvements and local electric grid characteristics. Under general case assumptions, the photovoltaic energy configuration was approximately 0.006 USD cap^–1^ day^–1^ higher than the grid-tied configuration but had lower GHG emissions by 23.6 kg CO_2_-equiv cap^–1^ year^–1^. The treatment units (sludge pasteurization, AnMBR, NCS, EC), controls, and photovoltaic power unit were the main drivers of the NEWgenerator cost for both energy sources, particularly due to component replacements and consumables. The NEWgenerator GHG emissions were primarily driven by the effluent, NCS, sludge pasteurization, and EC units. Direct emissions from dissolved methane and inefficient biogas combustion (resulting in fugitive methane) and life cycle emissions from production of consumables (LPG, chemicals) were the main contributors to emissions. The treatment performance, technical details, O&M requirements, and materials needed for construction were based on laboratory-based experiments and a 534-day South Africa field trial. The deployment of the NEWgenerator in other contexts was simulated by varying assumptions of influent composition, influent loading, electricity grid characteristics, and other contextual factors (e.g., LPG price), but other locality-specific factors (e.g., anal cleansing practices) will also be important to consider if more absolute costs and life cycle GHG emissions are required in specific deployment contexts. The sustainability indicators considered in this study were costs (economic) and life cycle GHG emissions (environmental), but future sustainability analyses may also consider the broader sanitation social-ecological system (S-SES) that defines sanitation technology as one component of a larger human-environmental system;^66^ additional studies on these aspects are critical for sustained use of NSS technologies.^50,65−69^

There are significant reductions in per capita cost which can be achieved by increasing the number of users without increasing the system’s physical footprint, but the potential of this approach is heavily dependent on whether the system can maintain treatment performance at these higher loadings and would require further laboratory and field testing. Targeted improvements to components (lithium photovoltaic battery, lower cost housing), reduced sludge moisture content, and frontend O&M ratio can provide significant cost and GHG reduction when combined. Further exploration of these alternative components, a low-cost sludge drying method prior to sludge pasteurization, and reducing O&M costs are recommended. The alternative zeolite management scenarios (replacement only and increased zeolite capacity) were more expensive and incurred more life cycle GHG emissions than the baseline design due to increased zeolite consumption and are not recommended. The addition of an HFMC for dissolved methane recovery is theoretically promising, with potential to significantly reduce GHG emissions and increase energy recovery with a modest increase in cost. Thus, the use of an HFMC with the NEWgenerator may be worth exploring experimentally. Resource recovery of nutrients at the current scale of users only had minimal reduction to per capita costs and life cycle GHG impacts through fertilizer offsets, but future work should explore the detailed design of resource recovery systems and mechanisms to improve economic outcomes of recovery at a larger scale of users. Resource recovery systems may target commercial quality fertilizers to enter existing fertilizer markets, but it is also important to note that the recovered resources may provide intangible benefits to the local community such as proximity to and lower costs of locally sourced fertilizers (to replace or supplement synthetic fertilizer products).

Contextual analysis through five country-specific simulations determined that the economic and environmental sustainability of the NEWgenerator was highly dependent on the context in which it will be deployed. The per capita cost of the NEWgenerator with photovoltaic energy across the five countries was as low as 0.068 USD cap^–1^ day^–1^ in India and as high as 0.100 USD cap^–1^ day^–1^ in Senegal, with higher costs for the grid-tied configuration ranging from 0.090 to 0.114 USD cap^–1^ day^–1^. The differences in per capita costs from the general case highlight the importance of contextual parameters in the characterization of the costs and life cycle GHG emissions of the NEWgenerator. The use of global average values may overestimate costs when deployment with locally manufactured or produced technologies is possible. Furthermore, subnational variations may lead to additional variations that can best be captured by more detailed consideration of specific localities under consideration for technology deployment.

Overall, the NEWgenerator has significant potential as a low-cost, low-GHG emission, and contextually flexible NSS system that can serve communities and informal settlements without sewered connections and regions where alternative onsite sanitation may not be feasible. Although the NEWgenerator has comparable costs and life cycle GHG emissions relative to other NSS technologies,^18,50^ sanitation systems reside in a broader social-ecological system that requires an understanding of how actors, governance systems, and related systems interact with the technology and determine its long-term success.^65^ Ultimately, the NEWgenerator shows promise as an NSS technology to address global sanitation gaps by providing safe, accessible sanitation across many contexts.

## Supporting Information Available

Detailed information on QSDsan input assumptions for NEWgenerator simulation; additional supporting system units (not part of the NEWgenerator); learning curve for scaled production; country-specific analysis; increase system capacity; Environmental impact assessment; uncertainty and sensitivity analysis; and targeted improvement scenarios (PDF)
