# Supplementary material for: Advancing the Economic and Environmental Sustainability of the NEWgenerator Nonsewered Sanitation System
Source: ACS Environ Au. 2023 May 5;3(4):209–22. doi: 10.1021/acsenvironau.3c00001 (PMC10360206; doi:10.1021/acsenvironau.3c00001)
Supplement: Supplementary file 1 — vg3c00001_si_001.pdf [file vg3c00001_si_001.pdf]

Supplementary Information for

Advancing the economic and environmental sustainability of the NEWgenerator^{TM} non-sewered sanitation system

Shion Watabe[a,b], Hannah A. C. Lohman[a], Yalin Li[c], Victoria L. Morgan[c], Lewis S. Rowles[c,d], Tyler Stephen[a], Hsiang-Yang Shyu[e], Robert A. Bair[e], Cynthia J. Castro[e], Roland D. Cusick[a], Daniel H. Yeh[e], Jeremy S. Guest[a,c,*]

Author Affiliations:

[a] Department of Civil and Environmental Engineering, University of Illinois Urbana-Champaign, 205 N. Mathews Ave., Urbana, Illinois, 61801, USA

[b] *Current Affiliation:* Stantec Australia Pty Ltd, Level 22, 570 Bourke Street, Melbourne, Victoria, 3000, AUSTRALIA

[c] Institute for Sustainability, Energy, and Environment, University of Illinois Urbana-Champaign, 1101 W. Peabody Dr., Urbana, Illinois, 61801, USA

[d] *Current Affiliation:* Department of Civil Engineering and Construction, Georgia Southern University, Statesboro, Georgia 30458, USA

[e] Department of Civil and Environmental Engineering, University of South Florida, 4202 E. Fowler Ave, Tampa, Florida, 33620, USA

*Corresponding author: jsguest@illinois.edu, +1 (217) 244-9247

Table of Contents (17 total pages):

Section S1. QSDsan input assumptions for NEWgenerator simulation

Section S2. Additional supporting system units (not part of the NEWgenerator)

Section S3. Learning curve for scaled production

Section S4. Country-specific analysis

Section S5. Increase system capacity

Section S6. Environmental impact assessment

Section S7. Uncertainty and sensitivity analysis

Section S8. Targeted improvement scenarios

Supporting References

Section S1. QSDsan input assumptions for NEWgenerator simulation

Table S1. Input waste characteristics for process simulation of the general case.

Parameter	Expected Value (range)	Distribution	Citation
Caloric Intake [$kcal \cdot cap^{-1} \cdot d^{-1}$]	2130 (1917-2343)	Uniform	[1]
Vegetal Protein Intake [$g \cdot cap^{-1} \cdot d^{-1}$]	40.29 (36.26-44.32)	Uniform	[1]
Animal Protein Intake [$g \cdot cap^{-1} \cdot d^{-1}$]	12.39 (11.15-13.63)	Uniform	[1]
Fraction of Nitrogen Content in Protein	0.13 (0.13-0.19)	Uniform	[2,3]
Fraction of Phosphorus in Vegetal Protein	0.022 (0.004-0.048)	Triangular	[2]
Fraction of Phosphorus in Animal Protein	0.011 (0.002-0.032)	Triangular	[2]
Potassium Content in Caloric Intake	1.2 (1.1-1.5)	Uniform	[4,5]
Fraction of Excreted Nitrogen in Intake	1 (0.99-1.0)	Uniform	[6,7]
Fraction of Excreted Phosphorus in Intake	1 (0.99-1.0)	Uniform	[6,7]
Fraction of Excreted Potassium in Intake	0.88 (0.65-0.98)	Uniform	[4,8]
Fraction of Energy in Intake	0.06 (0.02-0.10)	Uniform	[9–12]
Fraction of Nitrogen in Urine	0.88 (0.74-0.93)	Triangular	[9,10]
Fraction of Phosphorus in Urine	0.61 (0.33-0.75)	Triangular	[9,10]
Fraction of Potassium in Urine	0.74 (0.53-0.93)	Triangular	[9,10]
Fraction of Energy in Feces	0.81 (0.69-0.90)	Triangular	[9,10]
Fraction of Reduced Inorganic Nitrogen Reduced in Urine	0.85 (0.75-0.90)	Uniform	[9,10]
Fraction of Reduced Inorganic Nitrogen in Feces	0.20 (0.16-0.24)	Uniform	[10,13]
Urine Excretion [$g \cdot cap^{-1} \cdot d^{-1}$]	1400 (800-2500)	Triangular	[9,10]
Feces Excretion [$g \cdot cap^{-1} \cdot d^{-1}$]	250 (75-520)	Triangular	[9,10]
Fraction of Urine Moisture Content	0.95 (0.93-0.97)	Triangular	[9,10]
Fraction of Feces Moisture Content	0.85 (0.76-0.88)	Triangular	[9,10]
Magnesium in Urine [$g \cdot cap^{-1} \cdot d^{-1}$]	0.20 (0.12-0.21)	Uniform	[9,14]
Magnesium in Feces [$g \cdot cap^{-1} \cdot d^{-1}$]	0.25 (0.15-0.34)	Uniform	[9]
Calcium in Urine [$g \cdot cap^{-1} \cdot d^{-1}$]	0.28 (0.06-0.50)	Uniform	[9]
Calcium in Feces [$g \cdot cap^{-1} \cdot d^{-1}$]	1.90 (0.10-3.60)	Uniform	[9]

Table S2. Techno-economic analysis (TEA) assumptions used for discounted cash flow analysis.

Parameter	Expected Value (range)	Distribution	Citation
Discount rate [%]	5	-	[15]
Number of units for scaled production estimates	100,000	-	Service 10 million users
Learning curve percentage for scaling production cost – materials [%]	92.5 (90-95)	Uniform	[16]
Minimum cost limit [% of prototype cost]	2 (0-4)	Uniform	[17]
Price level ratio	0.25 (0.1 - 0.4)	Uniform	[18]

Table S3. NEWgenerator TEA input parameters for consumables, labor, and electricity in general case. Zeolite input parameter value obtained from Bill of Materials (BOM).

Parameter	Expected Value (range)	Distribution	Citation
Household electricity price [USD·kWh^{-1}]	0.06 (0.045-0.075)	Uniform	[19]
Maintenance labor wage [USD·h^{-1}]	3.64 (1.82-5.46)	Uniform	[20]
LPG price [USD·kg-LPG^{-1}]	1.523 (1.066-1.980)	Uniform	[21]
NaCl price [USD·kg-NaCl^{-1}]	0.276 (0.207-0.345)	Uniform	[22,23]
GAC price [USD·kg-GAC^{-1}]	1.10 (0.825-1.375)	Uniform	[24,25]
Zeolite price [USD·kg-Zeolite^{-1}]	0.23 (0.173-0.288)	Uniform	BOM

Table S4. NEWgenerator LCA input parameters for consumables, labor, and electricity in general case.

Parameter	Expected Value (range)	Distribution	Citation
Electricity intensity [kg CO_2eq·kWh^{-1}]	0.69 (0.62-0.76)	Uniform	[26]
Methane Characterization Factor [kg-CO_2eq·kg-$CH_4$$^{-1}$]	28 (28-34)	Uniform	[27]
N_2O Characterization Factor [kg-CO_2eq·kg- N_2O $^{-1}$]	265 (265-298)	Uniform	[27]
LPG Characterization Factor [kg-CO_2eq·kg-LPG^{-1}]	0.71 (0.50-0.92)	Uniform	[27]
NaCl Characterization Factor [kg-CO_2eq·kg-NaCl^{-1}]	0.27 (0.19-0.35)	Uniform	[27]
GAC Characterization Factor [kg-CO_2eq·kg-GAC^{-1}]	8.39 (5.87-10.91)	Uniform	[27]
Zeolite Characterization Factor [kg-CO_2eq·kg-Zeolite^{-1}]	5.18 (3.62-6.73)	Uniform	[27]

Section S2. Additional supporting system units (not part of the NEWgenerator)

Frontend

The frontend unit design consisted on an existing frontend toilet unit in QSDsan, assuming 1 seated toilet and 1 urinal per 25 people (4 seated toilets and 4 urinals for 100 users). In the increase system capacity scenario, the frontend was scaled up accordingly (**Section S5**). The frontend consisted of housing, seated toilet, urinal, fan, and miscellaneous parts (e.g., pipe, lighting, floor, etc.). The annual O&M cost was calculated as 7.5% of the total capital costs which would consist of replacements, labor, and maintenance. Direct methane and N_2O were from the collection of waste were calculated accordingly (**Section S6**).

Foundation

The foundation unit was based off of the concrete foundation pad design used to support the NEWgenerator in the India and South Africa field trials. A foundation area of 4.8m^2 and thickness of 0.1143 m (4.5 in) was implemented. It was assumed to have the same duration as the 25-year system lifetime and would not require any O&M.

Pretreatment

The pretreatment unit was based off of the pretreatment design in the South Africa field trial. It was consisted of the bar screen, pretreatment tank, piping, and feed pump. All components except the feed pump were assumed to have the same duration as the 25-year system lifetime, where feed pump had a 6-year lifetime according to the BOM. The O&M requirements consisted of pump replacements and labor associated with pump replacement and bar screen cleaning.

Sludge Pasteurization

The sludge pasteurization unit was designed to utilize the biogas and sludge produced from the AnMBR unit as a fuel source to treat the sludge on-site according to ISO 30500 solids output requirements. A scaled-down hydronic heat exchanger system and pump from an Omni Processor[28] was used to pasteurize the sludge and assumed to service 10 NEWgenerators, therefore factor of 0.1 was applied for economic and environmental analysis of one NEWgenerator. The pasteurization method heats the sludge by biogas combustion at 70 degrees Celcius for 30 minutes to achieve the LRV and maximum concentration requirements.[29] The process was assumed to have 90% biogas combustion efficiency or 10% biogas loss during the where the methane in the lost portion would be directly emitted to the atmosphere.[30] A 10% heat loss from the combustion heat transferred to the sludge was also assumed.[31]

S3. Learning curve for scaled production

A learning curve was used to conservatively estimate costs at scaled production. For the NEWgenerator, a production scale of 100,000 units was assumed. Each individual capital cost component or item, including Bill of Materials (BOM) specific items and additional items for the complete system, were assessed on its relevance to scaled serial production. The percentage of capital costs to be scaled from the total captail costs, was determined to be 65%. Indicating that 65% of the all capital cost items can be optimized with a learning curve as they could be mass-manufactured at a scaled production. The following generalized leaning curve function (Eq.1) was used to conservatively estimate the capital cost of the 100,000th unit produced and calculate a conservative estimate of user cost.

$$C_N = (C_1 - L)N^b + L \qquad \text{(Eq. 1)}$$

C_N is the cost of the Nth unit using the C_1, the first unit cost (which we assume to be the design team provided BOM or estimated cost at a single quantity), L the minimum cost limit, N the number of units, and b the learning curve exponent. assuming a certain rate of learning and efficiency improvement in the production process over time. Therefore, as the number of units increase the learning curve will approach the minimum cost limit asymptotically. A minimum cost limit of 1.5% of the first unit cost was assumed.[17,32] The learning curve exponent represents the rate of decrease in unit cost as additional units are manufactured (Eq. 2).

$$b = \frac{\log(learning\ curve\ percentage)}{\log(2)} \qquad \text{(Eq. 2)}$$

The learning curve percentage of 92.5% was assumed, which means that as the output doubles, the relative production cost is 92.5%. This means that the second unit costs 92.5% of the first unit, and then the fourth unit costs 92.5% of the second unit, which continues unit 100,00 units. This learning curve percentage of 92.5% for scaled material costs falls within the conservative range (typically 90-95%),[16] with lower values (e.g., 70%) is found to be aggressive. Learning curve percentages vary depending on the industry and type of production.

Section S4. Country-specific analysis

Due to the variety of different contextual economic and environmental factors, the user costs and GHG emissions for the NEWgenerator is dependent on the country of deployment. To capture these location-specific differences in the five countries of interest (China, India, South Africa, Senegal, and Uganda) in our analysis, input parameters were changed to reflect the expected conditions in each country. These country-specific parameters include household electricity price, energy mix GHG, price level ratio, tax rate, maintenance labor wage, vegetal protein, animal protein, caloric intake, food waste ratio, Liquified Petroleum Gas (LPG) price, and Sodium Chloride (NaCl) price. For specific parameters where country-specific data could not be obtained (e.g., maintenance labor wage in India, South Africa, and Senegal), we calculated and used the average from the countries with available data. The analysis provides insight into how deployment in specific countries which have unique local conditions can affect economic and environmental impact outcomes. It is important to note, however, that these results only reflect the 11 parameters examined and does not encompass the full set of conditions that may affect economic and environmental outcomes. The use of expected or average values for these countries of interest, in addition to data gaps in some parameters, only provides a high-level average potential outcome and does not capture variations that may occur in regions within these countries.

Table S5. Location-specific parameters used for contextual analysis for the NEWgenerator. When the specific value was not available, the general case value was used instead.[1,18–23,26]

Parameter	General Case	China	India	Senegal	South Africa	Uganda
Household electricity price [$USD\cdot kWh^{-1}$]	0.06	0.084	0.081	0.186	0.14	0.184
GHG intensity of grid electricity [$kg\ CO_2eq\cdot kWh^{-1}$]	0.69	0.745	0.852	0.939	0.955	0.159
Price level ratio	0.25	0.610	0.300	0.408	0.460	0.348
Maintenance labor wage [$USD\cdot h^{-1}$]	3.64	6.26	-	-	2.95	1.33
Vegetal protein [$g\cdot cap^{-1}\cdot d^{-1}$]	40.29	60.63	48.35	48.67	48.33	34.69
Animal protein [$g\cdot cap^{-1}\cdot d^{-1}$]	12.39	40	15	13.69	36.03	12.25
Caloric intake [$kcal\cdot cap^{-1}\cdot d^{-1}$]	2130	3191	2533	2545	2899	1981
LPG price [$USD\cdot L^{-1}$]	1.523	-	0.759	-	-	-
NaCl price [$USD\cdot kg\text{-}NaCl^{-1}$]	0.276	0.35	0.47	0.05	0.225	0.284

Table S6. The country-specific energy mixed used to determine the electricity GHG intensity for the contextual analysis.[26]

Energy source	General Case	China	India	Senegal	South Africa	Uganda
Fossil Fuel (Coal, Oil, Gas)	64%	69%	79%	88%	91%	6%
Hydro	25%	17%	9%	8%	0%	81%
Bio	3%	2%	3%	2%	0%	11%
Geo	1%	0%	0%	0%	0%	0%
Wind	3%	5%	4%	0%	3%	0%
Solar	2%	3%	3%	2%	2%	2%
Nuclear	3%	4%	2%	0%	4%	0%

Section S5. Increase System Capacity

Table S7. User scaling method and equations used for the increased NEWgenerator capacity simulation by category and example items.[33]

Category	Example Items	Scaling	Equation
Capital Costs	Pumps, electrochlorinator	Power	$NEWCAPEX = CAPEX\left(\frac{New\ Users}{100\ Users}\right)^{0.6}$
O&M Streams (consumables)	Zeolite, GAC, NaCl, NaOH	Linear	$NEW\ streams\ =\ streams\ wt.\frac{New\ Users}{100\ Users}$
O&M Replacements	Pumps, electrochlorinator	Power	$NEW\ OPEX = OPEX\left(\frac{New\ Users}{100\ Users}\right)^{0.6}$
Labor	Pump replacement	Linear	$NEW\ labor\ =\ labor\ hrs\frac{New\ Users}{100\ Users}$
Frontend	-	Linear	$\#\ Toilets = \frac{New\ Users}{25\ users\ per\ toilet}$
Electricity demand	Pump electricity consumption	Linear	$NEW\ kWh =\ kWh\frac{New\ Users}{100\ Users}$

The Sludge Pasteurization unit is an exception to the above user scaling category method where a linear scaling is utilized in addition to the sludge service assumption. This assumed that this unit will service 10 NEWgenerators, therefore factor of 0.1 was applied for economic and environmental analysis of one NEWgenerator.

Table S8. Items scaled for increased NEWgenerator capacity simulation in each unit and category.

Units	Capital Costs	O&M replacements	O&M Streams	Labor	Electricity Demand
Foundation	-	-	-	-	-
Ion exchange (NCS)	Pumps	Pumps	Zeolite, GAC, NaCl, NaOH	Pumps, Zeolite, GAC	Pumps
Housing	-	-	-	-	-
Pretreatment	Pump	Pump	-	Pump	Pump
AnMBR	Pumps, biogas storage	Pumps	-	Pump	Pumps
Sludge pasteurization	Hydronic heat exchanger, Pump	Hydronic heat exchanger, Pump	-	Hydronic heat exchanger, Pump	Hydronic heat exchanger, Pump
Photovoltaic	Photovoltaic panel, battery	Photovoltaic panel, battery	-	Battery	-
Grid	Power conversion, distribution box	-	-	-	-
Controls	-	-	-	-	-
Chlorination	Pumps, electrochlorinator	Pumps, electrochlorinator	NaCl	Pump	Pumps, electrochlorinator
Frontend (toilet)	Housing, toilets, urinals, piping, LED, floor, circuit change	-	-	-	-

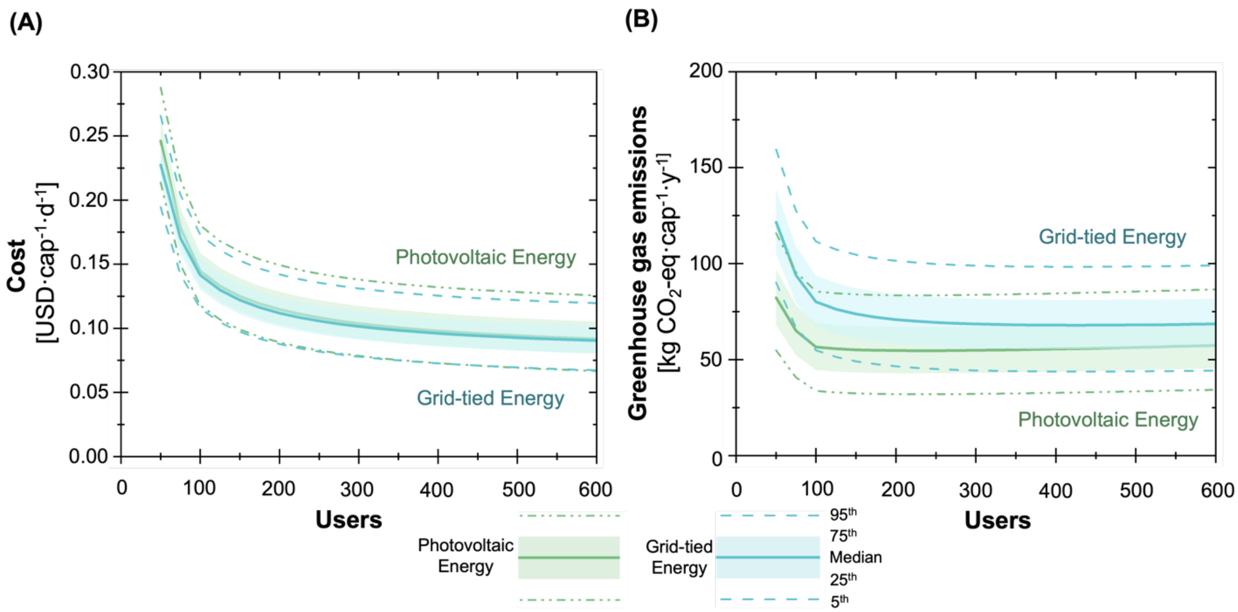

Figure S1. The daily user cost and annual user GHG emissions were simulated based on the impact of increasing users (increasing hydraulic throughput and loading rate) at NEWgenerator general case. Two different energy configurations were simulated: photovoltaic (green), and grid-tied (blue). The user capacity was simulated from 50 users to 600 users for user cost and emissions. The median, $25^{th}/75^{th}$, and $5^{th}/95^{th}$ are depicted by the solid line, shaded region, and dashed line, respectively, to represent the range of results from uncertainty analysis.

Section S6. Environmental impact assessment

Life cycle assessment (LCA) was performed to characterize the life cycle GHG emissions of the NSS system across the construction and operational stages. All sources of GHG emissions were normalized to global warming potential (GWP) with a functional unit of kg CO_2-eq·cap^{-1}·y^{-1}. The ecoinvent v3.2 database[27] was used to acquire inventory data for all materials and processes and translated to GWP using the U.S. EPA's Tool for the Reduction and Assessment of Chemicals and Other Environmental Impacts (TRACI 2.1 v1.03).[34] The emission sources from direct impacts from excreta, construction, electricity, and O&M impacts were calculated. Construction impacts were calculated for materials and processes of each component from the bill of materials (BOM), vendor websites, or manufacturers. If masses were not available from the BOM, vendors, or manufacturers the mass and materials for components were estimated and calculated using data or specifications available on dimensions, density, etc. Global or rest of world GHG emissions were used from the ecoinvent v3.2 database instead of country-specific for all analyses. Electricity impacts were calculated using the energy demand from the NEWgenerator BOM for the grid-tied configuration and the unit grid-electricity environmental impacts. The country-specific local grid electricity intensity was used for the contextual analysis. The energy produced from the photovoltaic configuration exceeded the NEWgenerator energy demand, therefore no environmental impacts from grid electricity were considered as it was not necessary. O&M impacts from consumables (production of) from the system processes and replacement components were calculated using the NEWgenerator maintenance schedule. Direct GHG emissions from excreta using during collection and treatment (e.g., user interface/frontend, containment, conveyance, pretreatment) were estimated. Direct GHG emissions of biogenic methane and N_2O released from bodily waste degradation and dissolved methane were considered in the frontend, effluent, and sludge pasteurization unit. Methane accounts for 28 times greater at 28 and N_2O approximately 265 times greater global warming impact than carbon dioxide[35,36] and was considered that way in emissions calculations (**Table S9**). For the process model, the direct methane and N_2O were calculated as a proportion of COD and nitrogen in the input waste stream. Dissolved methane remaining in the effluent after COD degradation in the anaerobic process was assumed to have direct GHG impact.

Table S9. Assumptions used for the calculation of direct methane and N_2O impact from excreta for lifecycle GHG emissions based on relevant literature.

Parameter	Expected Value (range)	Distribution	Citation
Maximum CH_4 emission [kg-CH_4·kg-COD^{-1}]	0.25 (0.175-0.325)	Triangular	[36,37]
Full degradation time	2 (1-3)	Uniform	[36,37]
Log degradation	3 (2-4)	Uniform	[36,37]
Nitrogen volatilization [fraction of N input]	0.005 (0.000-0.100)	Uniform	[13]
MCF decay [fraction of anaerobic conversion of degraded COD]	0.10 (0.05-0.15)	Triangular	[36,37]
N2O EF decay [fraction of N emitted as N2O]	0.0005 (0.000-0.001)	Uniform	[36,37]
Methane yield [m^3-CH_4·kg-COD^{-1}]	0.28 (0.23-0.33)	Uniform	[38]
Soluble methane fraction	0.30 (0.23-0.36)	Uniform	[39]

Section S7. Uncertainty and sensitivity analysis

The NEWgenerator QSDsan simulation involved 150 input parameters which values cannot be specified exactly (e.g., contextual differences, future change, data availability, numerous manufacturers). For each uncertain parameter a distribution (e.g., uniform, triangular) over a range was generated, where the range was defined using 15 – 35% variability based on accuracy and source of data or values. Latin Hypercube Sampling-based Monte Carlo simulation[40] was used for probabilistic generation of 10,000 sets of values for the uncertain parameters. A random seed of 5 was consistently used to generate the same number sequence and respective matching order of non-repeating 10,000 values for all input parameters in all simulations (e.g., photovoltaic configuration, grid-tied configuration). These input distributions were simulated to generate 10,000 output values which was used for the distributions of daily user cost and annual GHG emissions. Spearman's rank correlation coefficients were used to assess to the sensitivity of the results to the individual input parameters via the generated input and output distributions. The sensitivity was determined by how the functional output which is user cost or GHG emissions correlated to each individual input parameter by ranking the values in each input and their respective output distribution. Spearman's rank correlation coefficient determines the statistical dependence between the rankings of input parameter and output to assess the monotonic relationship. The correlation is high if the input parameter and output have a similar rank and low is dissimilar. The coefficient values range from -1 to 1, but for the purpose of this report absolute values of coefficients were used (0 to 1). The stronger the correlation between input parameter and output occurs with a larger the absolute value. The sensitivity analysis for input parameters by category (capital, O&M, direct) is detailed for daily user cost (**Figure S2**) and annual GHG emissions (**Figure S3**).

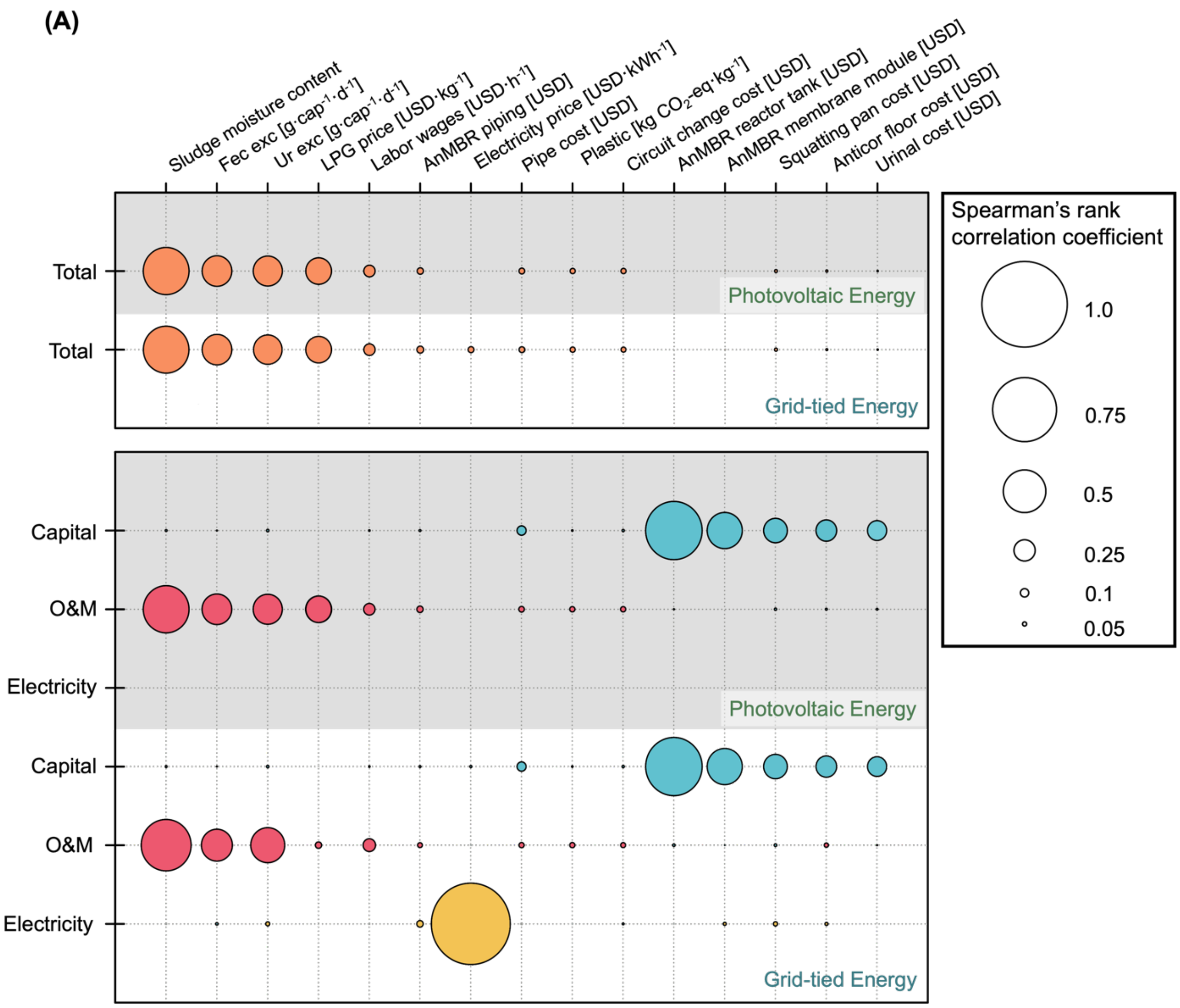

Figure S2. The Spearman's rank correlation coefficient for total, capital, and O&M (including labor, electricity, consumables, and component replacements) daily user cost for photovoltaic and grid-tied energy configurations. The size of the bubble indicates value of the correlation, with the larger bubble indicating higher correlation between the parameter and daily user cost which indicates relative sensitivity that the daily user cost has to the uncertainty of specific input parameter.

(B)

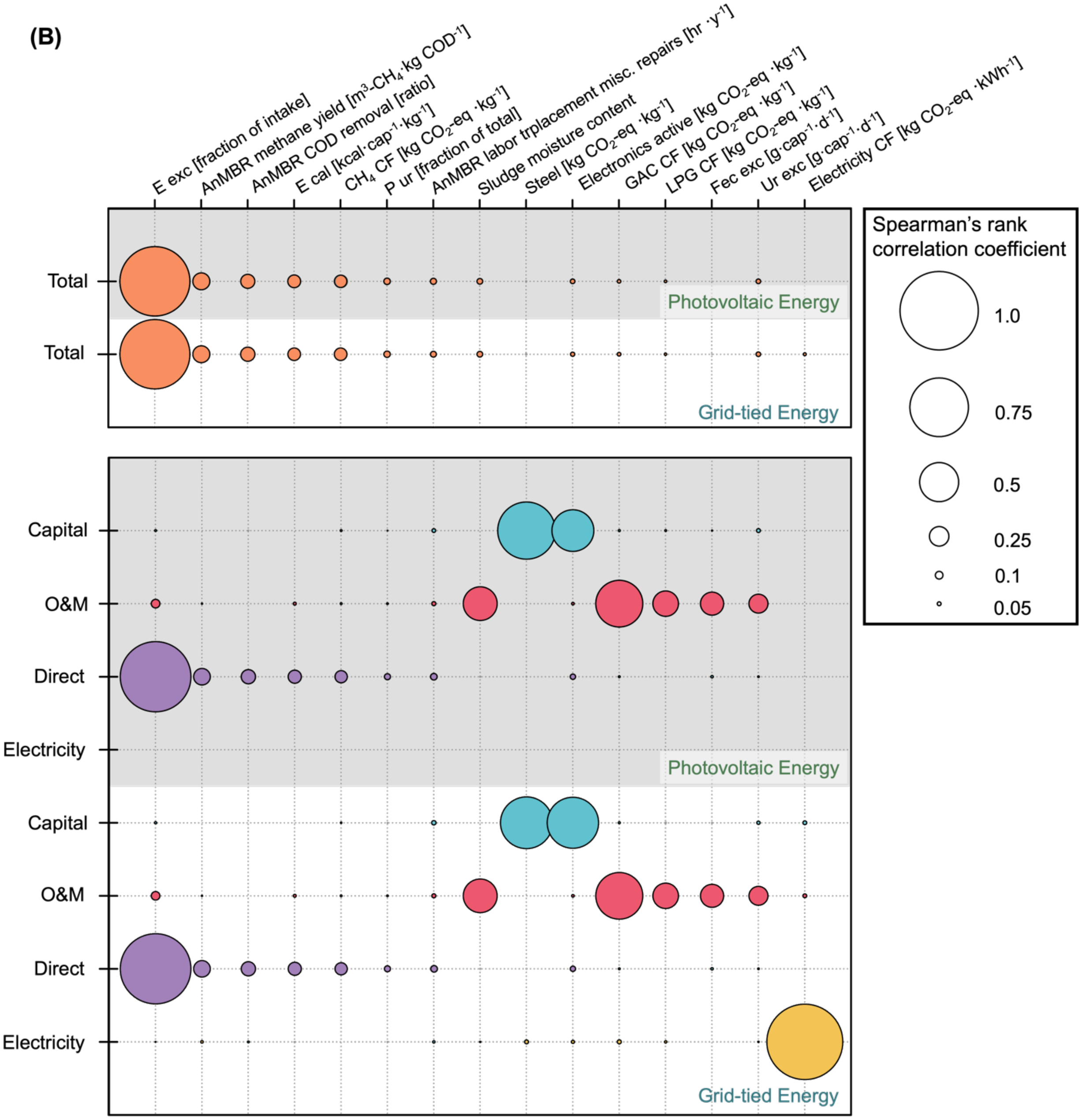

Figure S3. The Spearman's rank correlation coefficient for total, capital, O&M (including electricity, consumables, and component replacements), and direct annual GHG emissions for photovoltaic and grid-tied energy configurations. The size of the bubble indicates value of the correlation, with the larger bubble indicating higher correlation between the parameter and annual GHG emissions which indicates relative sensitivity that the annual GHG emissions has to the uncertainty of the specific input parameter.

S8. Targeted improvement scenarios

The targeted improvements (described in Scenario 2 through 4 of Methods) were simulated for the photovoltaic configuration NEWgenerator at general case for detailed breakdown (**Figure S4**) and differences (**Figure S5**). An additional scenario of LPG for sludge pasteurization only (no biogas) was explored, however resulted in negligible differences for user cost and GHG emissions from the baseline.

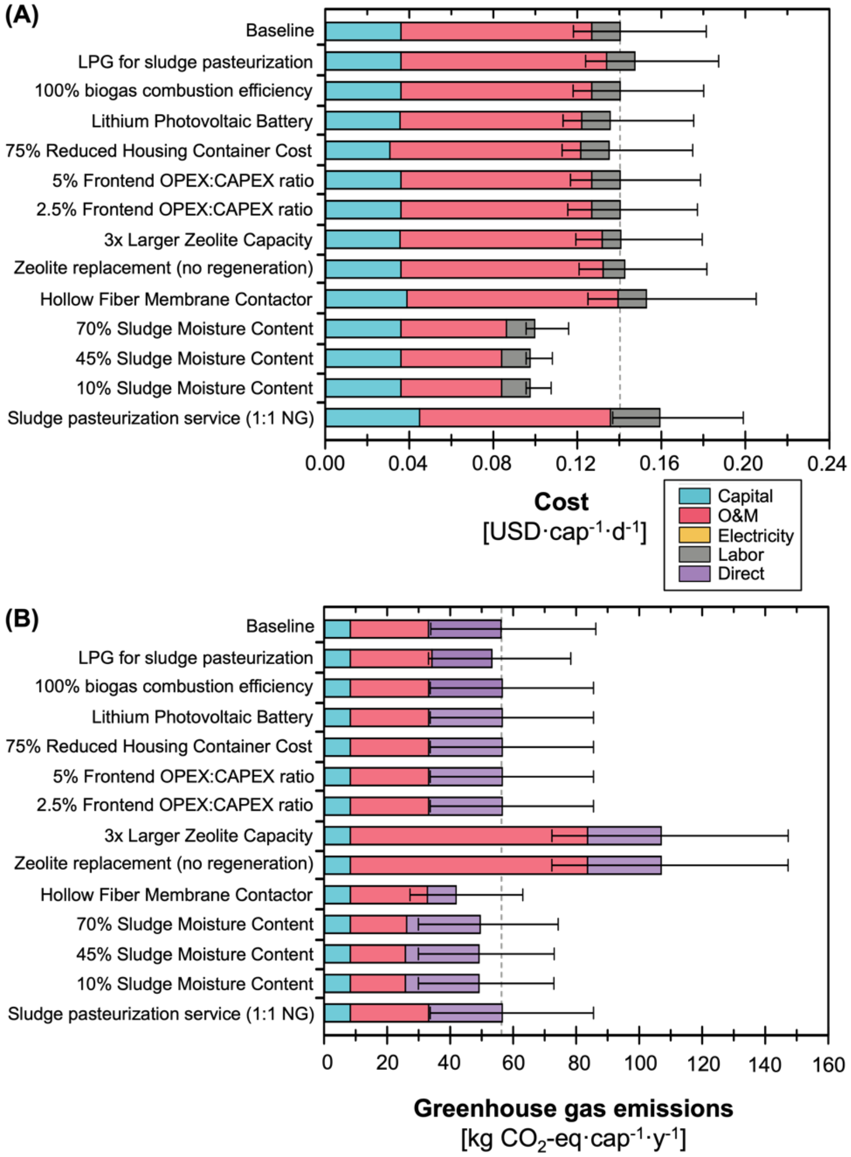

Figure S4. Targeted improvement scenarios compared to baseline for median user cost (A) and GHG emissions (B) at general case photovoltaic configuration NEWgenerator. The cost and emissions are broken down into the respective contributions of capital, O&M, electricity, labor, and direct (for GHG emissions only). The dashed vertical line indicated the baseline user cost of GHG emissions, and the stacked bars show the relative contributions from each source of cost and emissions. Error bars extend to the 5th and 95th percentile values from uncertainty analysis.

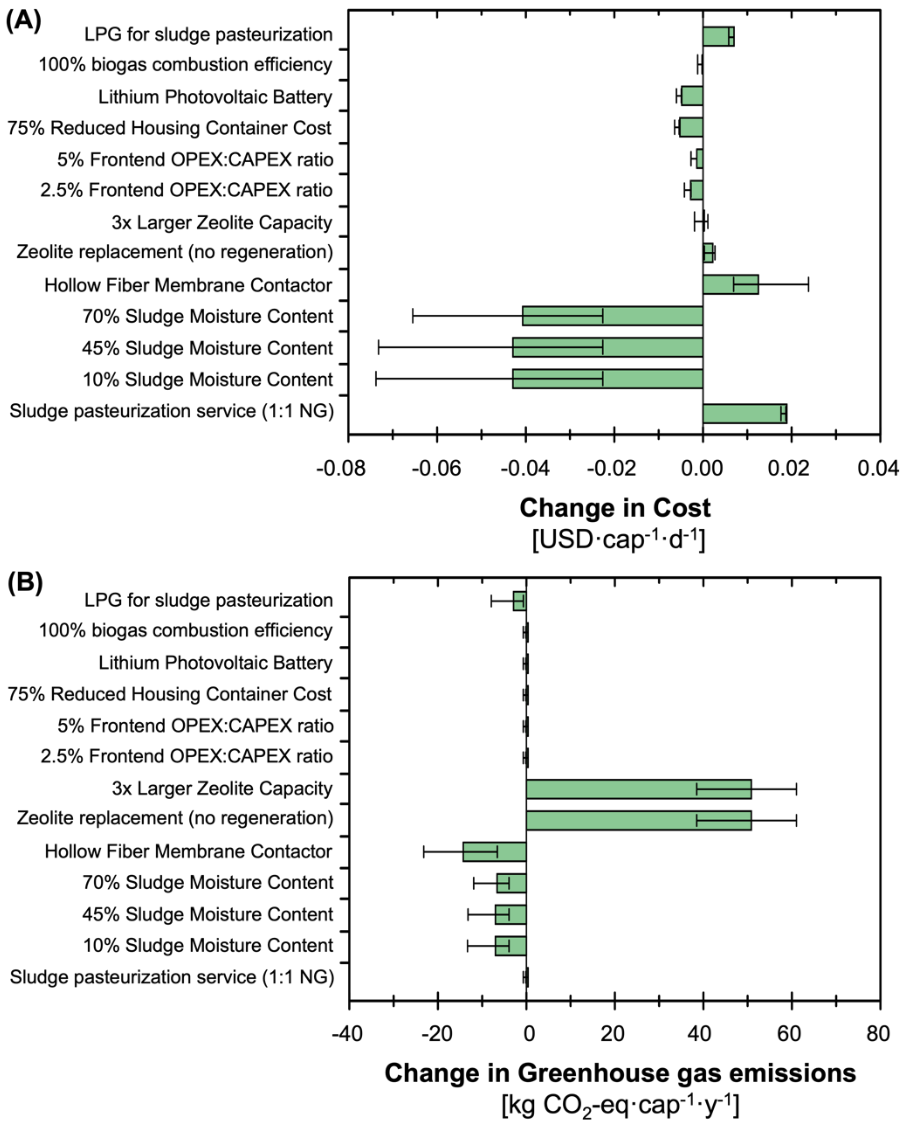

Figure S5. Targeted improvement scenarios differences from the baseline for median user cost (A) and GHG emissions (B) at general case photovoltaic configuration NEWgenerator. The value of zero is representative of the baseline median user cost of GHG emissions. Error bars extend to the 5th and 95th percentile difference values from uncertainty analysis.

Supporting References

(1) *FAOSTAT*. https://www.fao.org/faostat/en/#data/FBS (accessed 2022-05-04).

(2) Jonsson, H.; Stintzing, R.; Vinneras, B.; Salomon, E. *Guidelines on the Use of Urine and Faeces in Crop Production*; EcoSanRes Publications Series; Stockholm Environment Institute (SEI): Stockholm, Sweden, 2004; Vol. no. 2004-2.

(3) Food and Agriculture Organization of the United Nations. *Food Energy: Methods of Analysis and Conversion Factors: Report of a Technical Workshop, Rome, 3-6 December 2002*; MacLean, W. C., Warwick, P., Eds.; FAO food and nutrition paper; Food and Agriculture Organization of the United Nations: Rome, 2003.

(4) Holbrook, J. T.; Patterson, K. Y.; Bodner, J. E.; Douglas, L. W.; Veillon, C.; Kelsay, J. L.; Mertz, W.; Smith, J. C. Sodium and Potassium Intake and Balance in Adults Consuming Self-Selected Diets. *Am J Clin Nutr* **1984**, *40* (4), 786–793. https://doi.org/10.1093/ajcn/40.4.786.

(5) Kodama, N.; Morikuni, E.; Matsuzaki, N.; Yoshioka, Y. H.; Takeyama, H.; Yamada, H.; Kitajima, H.; Nishimuta, M. Sodium and Potassium Balances in Japanese Young Adults. *Journal of Nutritional Science and Vitaminology* **2005**, *51* (3), 161–168. https://doi.org/10.3177/jnsv.51.161.

(6) Rittmann, B. E.; Mayer, B.; Westerhoff, P.; Edwards, M. Capturing the Lost Phosphorus. *Chemosphere* **2011**, *84* (6), 846–853. https://doi.org/10.1016/j.chemosphere.2011.02.001.

(7) Richert, A.; R, G.; Jönsson, H.; Stenström, T.; L, D. *Practical Guidance on the Use of Urine in Crop Production.*; EcoSanRes series: 2010-1; SEI, 2010.

(8) Turban, S.; Miller, E. R.; Ange, B.; Appel, L. J. Racial Differences in Urinary Potassium Excretion. *J Am Soc Nephrol* **2008**, *19* (7), 1396–1402. https://doi.org/10.1681/ASN.2007101142.

(9) Rose, C.; Parker, A.; Jefferson, B.; Cartmell, E. The Characterization of Feces and Urine: A Review of the Literature to Inform Advanced Treatment Technology. *Crit Rev Environ Sci Technol* **2015**, *45* (17), 1827–1879. https://doi.org/10.1080/10643389.2014.1000761.

(10) Friedler, A.; Butler, D.; Alfiya, Y. Wastewater Composition. In *Source Separation and Decentralization for Wastewater Management*; IWA Publishing: London, UK, 2013; pp 241–258.

(11) McCarty, P. L.; Bae, J.; Kim, J. Domestic Wastewater Treatment as a Net Energy Producer–Can This Be Achieved? *Environ. Sci. Technol.* **2011**, *45* (17), 7100–7106. https://doi.org/10.1021/es2014264.

(12) Hall, K. D.; Heymsfield, S. B.; Kemnitz, J. W.; Klein, S.; Schoeller, D. A.; Speakman, J. R. Energy Balance and Its Components: Implications for Body Weight Regulation123. *Am J Clin Nutr* **2012**, *95* (4), 989–994. https://doi.org/10.3945/ajcn.112.036350.

(13) Orner, K. D.; Mihelcic, J. R. A Review of Sanitation Technologies to Achieve Multiple Sustainable Development Goals That Promote Resource Recovery. *Environ. Sci.: Water Res. Technol.* **2017**, *4* (1), 16–32. https://doi.org/10.1039/C7EW00195A.

(14) Udert, K. M.; Larsen, T. A.; Gujer, W. Biologically Induced Precipitation in Urine-Collecting Systems. *Water Supply* **2003**, *3* (3), 71–78. https://doi.org/10.2166/ws.2003.0010.

(15) Haacker, M.; Hallett, T. B.; Atun, R. On Discount Rates for Economic Evaluations in Global Health. *Health Policy and Planning* **2020**, *35* (1), 107–114. https://doi.org/10.1093/heapol/czz127.

(16) Yelle, L. E. The Learning Curve: Historical Review and Comprehensive Survey. *Decision Sciences* **1979**, *10* (2), 302–328. https://doi.org/10.1111/j.1540-5915.1979.tb00026.x.

(17) Wong, L. F. A Generalized Learning Curve Adapted for Purchasing and Cost Reduction Negotiations. *Advances in Operations Research* **2013**, *2013*, e584762. https://doi.org/10.1155/2013/584762.
(18) *Price level ratio of PPP conversion factor (GDP) to market exchange rate | Data*. https://data.worldbank.org/indicator/pa.nus.pppc.rf?view=map (accessed 2022-05-04).
(19) *Electricity prices around the world*. GlobalPetrolPrices.com. https://www.globalpetrolprices.com/electricity_prices/ (accessed 2022-05-04).
(20) *Statistics on Wages*. ILOSTAT. https://ilostat.ilo.org/topics/wages/ (accessed 2022-05-04).
(21) *LPG prices around the world, 02-May-2022*. GlobalPetrolPrices.com. https://www.globalpetrolprices.com/lpg_prices/ (accessed 2022-05-04).
(22) *Salt - prices around the world, June 2022 | GlobalProductPrices.com*. https://www.globalproductprices.com/rankings/salt_prices/ (accessed 2022-08-16).
(23) *Salt prices U.S. by type 2021*. Statista. https://www.statista.com/statistics/916733/us-salt-prices-by-type/ (accessed 2022-08-16).
(24) Mukherjee, A.; Okolie, J. A.; Niu, C.; Dalai, A. K. Techno – Economic Analysis of Activated Carbon Production from Spent Coffee Grounds: Comparative Evaluation of Different Production Routes. *Energy Conversion and Management: X* **2022**, *14*, 100218. https://doi.org/10.1016/j.ecmx.2022.100218.
(25) *Activated carbon market value 2029*. Statista. https://www.statista.com/statistics/1310445/activated-carbon-market-value-worldwide/ (accessed 2022-08-16).
(26) *International - U.S. Energy Information Administration (EIA)*. https://www.eia.gov/international/data/world/electricity/electricity-generation (accessed 2022-05-04).
(27) Ecoinvent 3.2 Database. Swiss Centre for Life Cycle Inventories 2016.
(28) Rowles, L. S.; Morgan, V. L.; Li, Y.; Zhang, X.; Watabe, S.; Stephen, T.; Lohman, H. A. C.; DeSouza, D.; Hallowell, J.; Cusick, R. D.; Guest, J. S. Financial Viability and Environmental Sustainability of Fecal Sludge Treatment with Pyrolysis Omni Processors. *ACS Environ. Au* **2022**. https://doi.org/10.1021/acsenvironau.2c00022.
(29) US EPA, O. *Control of Pathogens and Vector Attraction in Sewage Sludge*. https://www.epa.gov/biosolids/control-pathogens-and-vector-attraction-sewage-sludge (accessed 2021-12-06).
(30) Voegeli, Y. *Anaerobic Digestion of Biowaste in Developing Countries: Practical Information and Case Studies*; Eawag-Sandec: Dübendorf, 2014.
(31) D. Shoener, B.; Zhong, C.; D. Greiner, A.; Khunjar, W. O.; Hong, P.-Y.; S. Guest, J. Design of Anaerobic Membrane Bioreactors for the Valorization of Dilute Organic Carbon Waste Streams. *Energy & Environmental Science* **2016**, *9* (3), 1102–1112. https://doi.org/10.1039/C5EE03715H.
(32) Linton, J. D.; Walsh, S. T. Integrating Innovation and Learning Curve Theory: An Enabler for Moving Nanotechnologies and Other Emerging Process Technologies into Production. *R&D Management* **2004**, *34* (5), 517–526. https://doi.org/10.1111/j.1467-9310.2004.00359.x.
(33) Cortes-Peña, Y.; Kumar, D.; Singh, V.; Guest, J. S. BioSTEAM: A Fast and Flexible Platform for the Design, Simulation, and Techno-Economic Analysis of Biorefineries under Uncertainty. *ACS Sustainable Chem. Eng.* **2020**, *8* (8), 3302–3310. https://doi.org/10.1021/acssuschemeng.9b07040.
(34) Bare, J. TRACI 2.0: The Tool for the Reduction and Assessment of Chemical and Other Environmental Impacts 2.0. *Clean Techn Environ Policy* **2011**, *13* (5), 687–696. https://doi.org/10.1007/s10098-010-0338-9.

(35) *AR5 Synthesis Report: Climate Change 2014 — IPCC*. https://www.ipcc.ch/report/ar5/syr/ (accessed 2022-06-21).
(36) 2019 Refinement to the 2006 IPCC Guidelines for National Greenhouse Gas Inventories — IPCC.
(37) Eggleston, H. S.; Buendia, L.; Miwa, K.; Ngara, T.; Tanabe, K. 2006 IPCC Guidelines for National Greenhouse Gas Inventories. **2006**.
(38) Abuabdou, S. M. A.; Ahmad, W.; Aun, N. C.; Bashir, M. J. K. A Review of Anaerobic Membrane Bioreactors (AnMBR) for the Treatment of Highly Contaminated Landfill Leachate and Biogas Production: Effectiveness, Limitations and Future Perspectives. *Journal of Cleaner Production* **2020**, *255*, 120215. https://doi.org/10.1016/j.jclepro.2020.120215.
(39) Kim, J.; Kim, K.; Ye, H.; Lee, E.; Shin, C.; McCarty, P. L.; Bae, J. Anaerobic Fluidized Bed Membrane Bioreactor for Wastewater Treatment. *Environ Sci Technol* **2011**, *45* (2), 576–581. https://doi.org/10.1021/es1027103.
(40) Schmidt, R.; Voigt, M.; Mailach, R. Latin Hypercube Sampling-Based Monte Carlo Simulation: Extension of the Sample Size and Correlation Control. In *Uncertainty Management for Robust Industrial Design in Aeronautics : Findings and Best Practice Collected During UMRIDA, a Collaborative Research Project (2013–2016) Funded by the European Union*; Hirsch, C., Wunsch, D., Szumbarski, J., Łaniewski-Wołłk, Ł., Pons-Prats, J., Eds.; Notes on Numerical Fluid Mechanics and Multidisciplinary Design; Springer International Publishing: Cham, 2019; pp 279–289. https://doi.org/10.1007/978-3-319-77767-2_17.
